# Quorum-sensing regulator LsrR modulates avian pathogenic *Escherichia coli* pathogenicity through direct regulation of *cysN*

**DOI:** 10.1128/iai.00421-25

**Published:** 2025-11-28

**Authors:** Saqib Nawaz, Zhihao Wang, Wei Jiang, Lanfang Kong, Huifang Yin, Yinli Bao, Cuiqin Huang, Zhaoguo Chen, Yan Zhang, Xiangan Han

**Affiliations:** 1 Shanghai Veterinary Research Institute, Chinese Academy of Agricultural Sciences, Minhang 118161 , Shanghai, People's Republic of China; 2 Engineering Research Center for the Prevention and Control of Animal Original Zoonosis, Fujian Province, College of Life Science, Longyan University 481554, Longyan, Fujian, China; Stanford University School of Medicine, Stanford, California, USA

**Keywords:** virulence regulation, sulfur metabolism, immune evasion, efflux pumps, host-pathogen interaction

## Abstract

Avian Pathogenic *Escherichia coli* (APEC) is a major cause of economic loss in poultry, exacerbated by the rising prevalence of antibiotic resistance. While sulfur metabolism is essential for bacterial growth, its specific role and regulation in APEC virulence remain poorly understood. This study identifies the LsrR-cysN axis as a novel regulatory pathway that critically governs APEC virulence. We demonstrate that the quorum-sensing regulator LsrR directly binds to the *cysN* promoter, activating its transcription. Functional analysis revealed that *cysN* deletion drastically attenuated virulence, significantly reducing biofilm formation, serum resistance, adhesion, invasion, and motility. The APEC94∆cysN also exhibited altered antibiotic resistance profiles, which were linked to the upregulation of efflux pumps *acrA* and *tolC*. Crucially, in a murine model, the APEC94∆cysN showed a 75% reduction in mortality and severe impairment in colonization of blood, lungs, liver, spleen, and kidneys. This attenuation was associated with a skewed host immune response, characterized by reduced levels of IL-2 and IL-6 and elevated levels of IL-4 and TNF-α. Our findings establish the LsrR-cysN axis as a central regulator connecting quorum sensing to virulence in APEC, revealing a promising target for novel anti-virulence strategies.

## INTRODUCTION

Avian Pathogenic *Escherichia coli* (APEC) is a prototype extraintestinal pathogenic *E. coli* that, despite often originating from the gastrointestinal tract microbiota, can cause severe respiratory and systemic infections through complex host-pathogen interactions (1). The pathogenic potential of APEC is closely linked to its ability to acquire virulence genes (VGs) that facilitate adherence, invasion, the establishment of secretion systems, toxin production, modulation of the immune response, biofilm development, and various metabolic processes. APEC infections lead to substantial economic losses due to increased mortality, decreased productivity, and costs related to antibiotic use. Annually, there is a $40 million financial loss to the broiler industry due to carcass condemnation alone in the United States. Similarly, a €3.7 million loss is incurred due to APEC infections in poultry farms in the Netherlands. In Indonesia, the estimated loss was $1.049 billion and $992 million per harvest for broilers and layers, respectively (2). Understanding APEC’s virulence factors is essential for developing effective control measures, as APEC poses a zoonotic risk and may contribute to the global spread of antibiotic resistance (3).

The *cysN* gene encodes a subunit of the ATP sulfurylase enzyme, which catalyzes the conversion of inorganic sulfate (SO_4_²^-^) into adenosine-5′-phosphosulfate, initiating sulfate activation (4). This pathway is essential for cysteine synthesis, a critical sulfur-containing amino acid, which plays several vital roles in APEC survival and pathogenicity (e.g., combating reactive oxygen species [ROS], cellular protection, maintaining redox balance [5], and enhancing bacterial replication [6]). Notably, the importance of cysteine biosynthesis for virulence is a conserved theme in other pathogens, where these genes contribute to oxidative stress resistance in *Salmonella typhimurium* and persistence in *Mycobacterium tuberculosis* (7, 8). This makes *cysN* a critical factor for APEC survival and pathogenicity in host environments (9).

Beyond metabolic pathways, virulence in APEC is also controlled by global regulatory systems, such as quorum sensing. The QS regulator LsrR is essential for the pathogenicity of APEC as it modulates QS signals that influence the expression of virulence factors (10, 11). It governs the uptake and processing of autoinducers, essential molecules facilitating inter-bacterial communication (12). Our preliminary data showed that LsrR binds the *cysN* promoter and that this interaction is specifically responsive to phospho-AI-2 *in vitro*, indicating that LsrR directly recognizes this signal (13). The LsrR selection for binding experiments with *cysN* is predicated on the assumption that LsrR serves as a regulatory protein that may impact sulfur metabolism, particularly in light of *cysN* involvement in cysteine biosynthesis (10). The significance of LsrR is underscored by its distinctive capacity to adjust gene expression in response to QS signals, potentially influencing a range of metabolic pathways, including those associated with *cysN* (14). Furthermore, other proteins may lack comparable regulatory importance, rendering LsrR a more appropriate candidate for investigating interactions (10, 12). The flagellar, type 1 fimbrial, and virulence genes in gram-negative pathogens enable bacteria to deliver effector proteins directly into host cells (15). These genes bypass the host’s defense mechanisms, disrupt cellular processes, and facilitate bacterial survival and infection (14). Additionally, they undermine the host’s innate immunity (15) and promote pathogen colonization (16). Collectively, LsrR and *cysN* enhance APEC’s capacity to colonize, endure, and multiply within host tissues (14, 17).

The role of *cysN* in the cysteine biosynthesis pathway is posited to impact bacterial metabolism (18). It is hypothesized that *cysN* contributes to APEC pathogenic processes, such as flagellar motility (19) expression of type-1 fimbriae, regulation of virulence genes, bacterial motility, serum resistance, and colonization abilities (20). By investigating these factors, the objective is to elucidate the role of *cysN* in APEC pathogenicity and its influence on the APEC capacity to adapt, survive, and initiate infections, which are essential characteristics for virulence and persistence within host environments (21).

## RESULTS

### Expression of the LsrR protein

**Fig 1:**
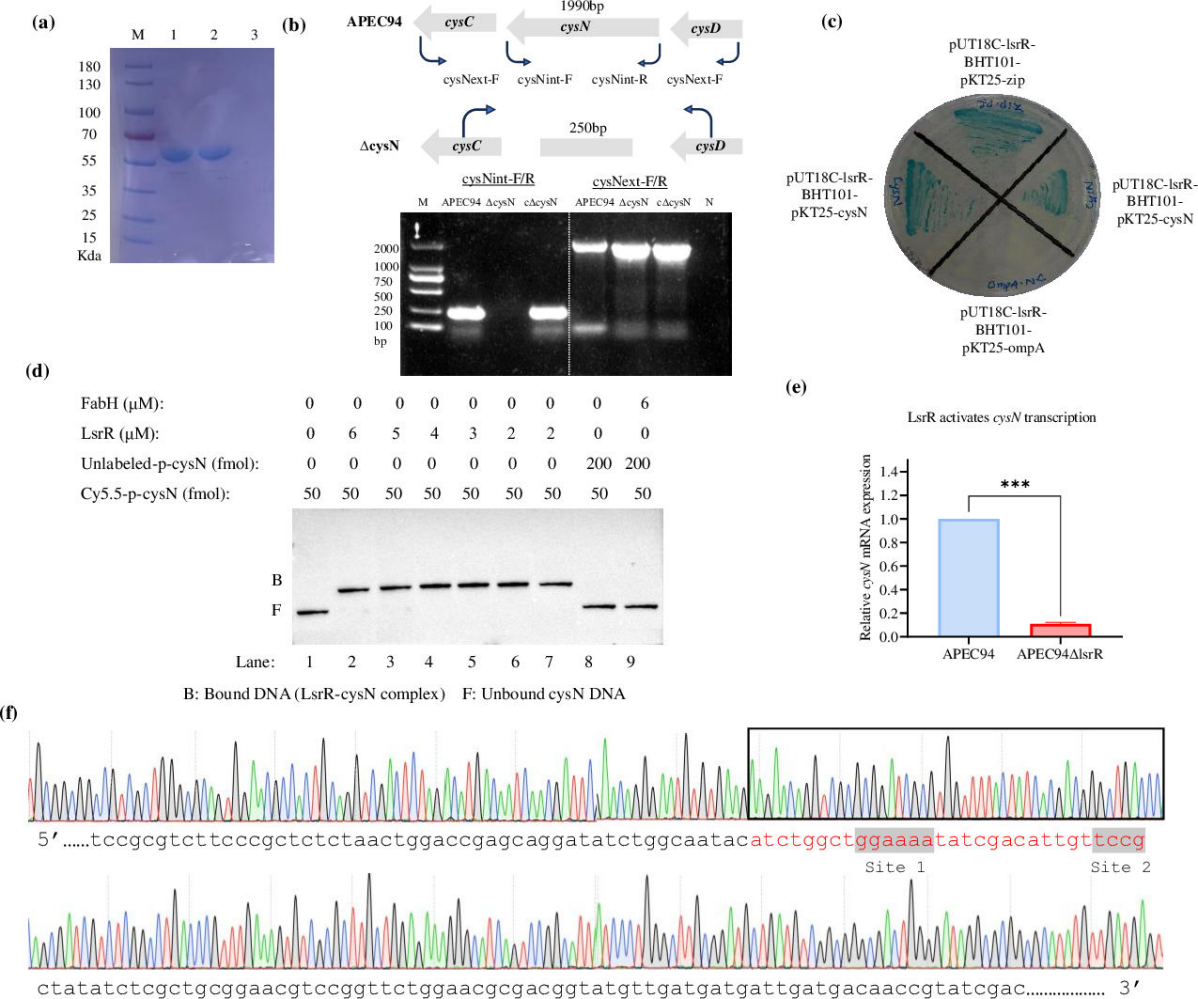
(**a**) The LsrR protein was expressed and purified. M: BlueStar Prestained Protein Marker (180 kDa); Line 1: purified LsrR protein flow-through with a size of 60 kDa; Line 2: LsrR positive control; and Line 3: PBS negative control. (**b**) Graphic representation and confirmation of the mutant and complementary strains by PCR. PCR lanes 1–3 confirmed *cysN* deletion with the *cysN* internal primer-F/R, and lanes 4–6 confirmed *cysN* deletion with the *cysN* external primer-F/R. The primers used for the PCR identification of the *cysN* gene deletion are also indicated. The amplification of the corresponding target genes indicates success in constructing mutant and complemented strains. (**c**) Bluish colonies on X-gal plates indicated interaction between LsrR and cysN (pUT18C-lsrR-BHT101-pKT25-cysN). In contrast, pUT18C-lsrR-BHT101-pKT25-ompA was used as a negative control (no interaction/whitish colonies) and pUT18C-lsrR-BHT101-pKT25-zip as a positive control (interaction/ bluish colonies). (**d**) LsrR binds to the *cysN* promoters. The binding ability of LsrR to the cysN promoters was determined by electrophoretic mobility shift assay. An increasing amount of LsrR was incubated with excess Cy5.5-labeled probes. Lane 1: free probe. In each panel, from lanes 2 to 7, the concentration of LsrR was 6, 5, 4, 3, 2, and 2 μM, respectively; the amount of Cy5.5-labeled probes was all 50 fmol (the concentrations were all 5 nM). In lanes 8–9, 200 fmol of unlabeled probes were incubated with the LsrR protein beside the labeled probes. In lane 9, protein FabH was run as a negative control. (**e**) LsrR activates *cysN* transcription. Relative expression of the *cysN* gene in the APEC94 strain compared to the APEC94∆lsrR mutant, as determined by qRT-PCR. Gene expression was normalized to the housekeeping gene *dnaE*, and the relative expression level in the APEC94 was set to 1. Data are presented as the mean ± SEM from three independent biological replicates. Statistical significance was determined by an unpaired *t*-test (***P < 0.001). (**f**) DNase I footprinting identified an LsrR-binding site within the *cysN* promoter. Sequence alignment of this protected region revealed the LsrR conserved binding sequence.

The expression and purification of the LsrR protein were carried out by cloning the *lsrR* gene (PCR amplified from *E. coli* genomic DNA) into an expression plasmid, i.e., pGEX-4T-1 series for *E. coli*, and IPTG-inducible expression with a GST-tag for purification. The *lsrR* gene was expressed in the BL21(DE3) strain for T7 polymerase-driven expression, as shown in Fig. 1a.

### Construction of the mutant APEC94∆cysN and the complementary strain

The *cysN* gene mutant and complemented strains of APEC94 were constructed and confirmed by PCR amplification using respective primers. The PCR and sequencing validated the successful construction of mutant and complemented strains. Plasmids pTarget and pstv28 were utilized for constructing mutant APEC94∆cysN and complementary cAPEC94∆cysN strains. Vector-only controls did not alter phenotype, confirming that complementation effects were attributable to the reintroduction of *cysN* rather than plasmid carriage. The *cysN* gene, located between *cysC* and *cysD*, has a set base size of 1,990 bp, determined by designing and validating the appropriate primers. The mutants were confirmed by internal and external detection primers, such as cysNext-F, cysNext-R, cysNint-F, and cysNint-R. ∆cysN indicates the mutant APEC94∆cysN, and c∆cysN indicates the complementary cAPEC94∆cysN strains (Fig. 1b). The X-gal (5-bromo-4-chloro-3-indolyl-β-D-galactopyranoside) LB plates detected LsrR binding to its target promoter. The resulting bluish colonies on X-gal LB agar plates determined that the recombinant plasmids pUT18C-LsrR and pKT25-cysN interacted. pUT18C-lsrR and pKT25-zip interacted, resulting in bluish colonies, serving as a positive control, whereas pUT18C-lsrR and pKT25-ompA resulted in colorless colonies, serving as a negative control (Fig. 1c).

### LsrR binds to the *cysN* promoters

The DNA fragment p-cysN (131 bp, from −1 to −131 of the cysN translation start site) was PCR-amplified and labeled with Cy5.5 for use as a probe. The results indicate that LsrR binds specifically to the *cysN* promoter regions. As illustrated in Fig. 1d, two bands are identified from lanes 1 to 8. The lower band shows free labeled probes in molar excess, while the upper band represents a protein-DNA complex. The amount of LsrR protein was increased from 0 to 60 pmol (final concentration 0 to 6 µM) (right to left), resulting in a corresponding decrease in the amount of free probes. No LsrR was added to lane 1 for control, and 10-fold unlabeled fragments were used as specific competitors in lane 8; however, no protein-DNA complex formed under these conditions. In lane 9, the FabH protein served as a negative control, and the results revealed that FabH does not bind with Cy5.5-labeled cysN. These results demonstrate that LsrR can bind to the *cysN* promoter regions *in vitro*.

### LsrR activates *cysN* transcription

Having established that LsrR binds specifically to the *cysN* promoter *in vitro*, we determined the functional consequence of this interaction under defined laboratory growth conditions. We constructed APEC94∆lsrR (a *lsrR* deletion mutant) and used qRT-PCR to quantify *cysN* transcript levels in the APEC94 and APEC94∆lsrR during the mid-logarithmic growth phase in LB broth. The deletion of *lsrR* resulted in a significant decrease in *cysN* mRNA levels. Compared to the APEC94, the APEC94∆lsrR mutant exhibited a 5.2-fold reduction in *cysN* transcription (*P* < 0.001; Fig. 1e). This finding demonstrates that LsrR functions as a positive regulator, activating the transcription of the *cysN* gene *in vitro*.

### Identification of the LsrR-binding sites in the *cysN* promoters

A DNase I footprinting was performed to identify the precise LsrR-binding sequence. A fragment incorporating the +41 to –114 region relative to the translation start site of the *cysN* gene was fluorescently 5′-labeled with Cy5.5 and incubated with increasing amounts of GST-tag-LsrR before being treated with DNase I. These regions represent the usual binding sites of transcription factors (22). The digestion patterns were examined by capillary electrophoresis (CE) using a 3730XL automated sequencer (Applied Biosystems). The results of the CE were analyzed using Genemapper V3.5 software, which enabled the precise identification of the LsrR-binding sequence. As shown in Fig. 1f, the LsrR conserved binding sequence was identified through sequence alignment of the p-cysN-box using Vector NTI Suite 9 software. A black border indicates the regions of conservative sites. LsrR binds to a 30 bp region (ATCTGGCTGGAAAATATCGACATTGTTCCG) of the *cysN* promoter region, which is positioned just within the −289 to −319 promoter box relative to the translation start site.

### Sequence analysis of the LsrR-binding box

Two sequences in the p-cysN-box, a 4 bp sequence (TCCG) and a 6 bp sequence (GGAAAA), were determined to be important for LsrR binding based on the sequence alignment results, which show that both sequences are generally conserved (23). By generating the appropriate deletions or mutations, several variants of pPcysN were constructed (Fig. S4a).

### Phenotypic assays of the mutated promoters of *cysN*

The findings indicate that LsrR cannot bind to the *cysN* promoter when the 4 or 6 bp sequences are deleted or mutated, resulting in a decrease in binding affinity. Promoters with site-specific mutations (random GCs were substituted for the original 4 to 6 bp AT-rich segments) were constructed. The site-deleted/mutated Cy5.5-labeled *cysN* probe was also run on the EMSA gel to confirm that no LsrR protein bound to the *cysN* gene promoter because of the deletion and mutation of binding sites (Fig. S4b and c).

### *cysN* deletion affects the biological characteristics of APEC94

**Fig 2:**
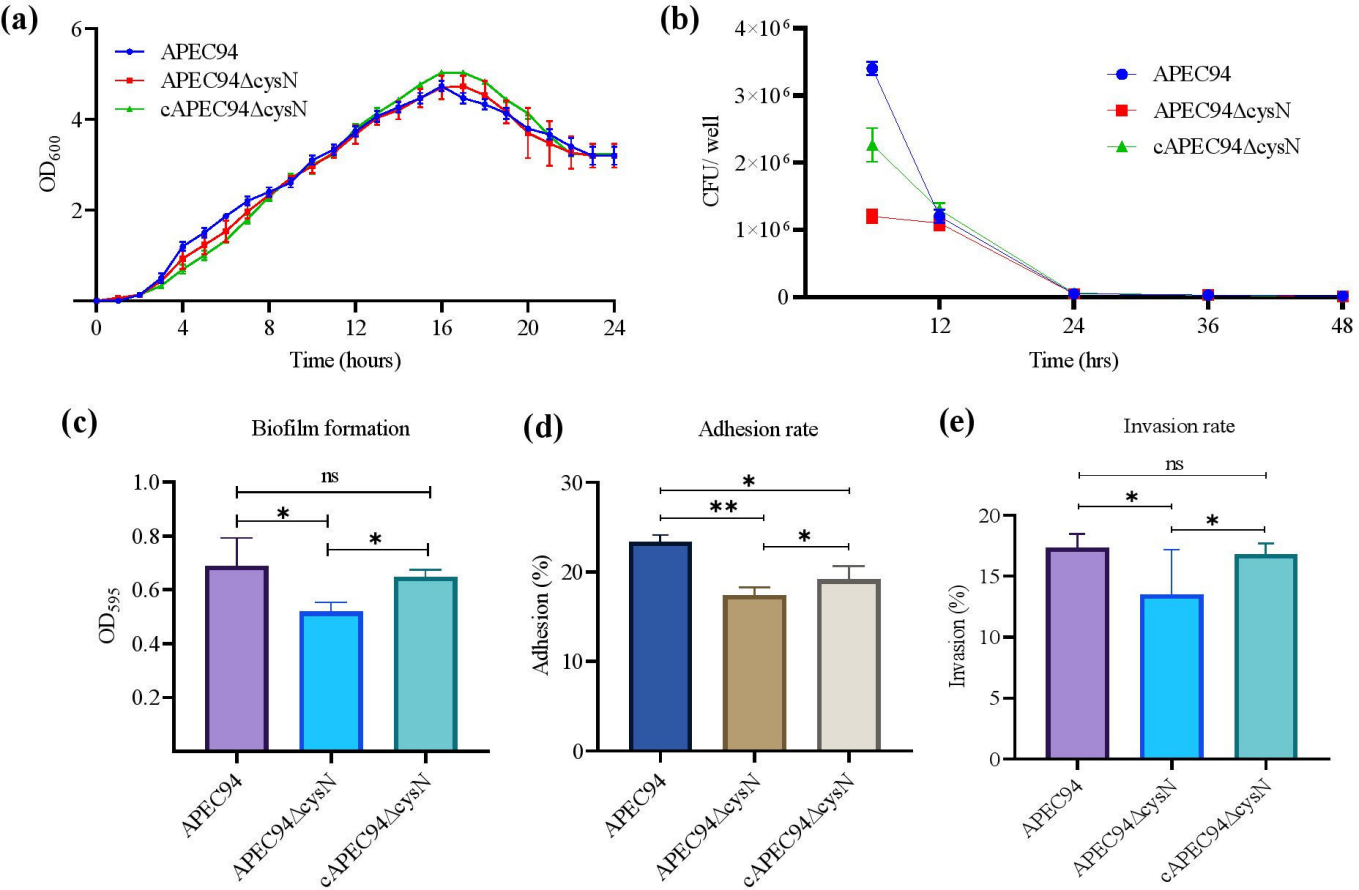
Biological characteristics analysis of the three APEC94 strains. (**a**) Growth curves of APEC94, APEC94∆cysN, and cAPEC94∆cysN strains in LB. (**b**) RAW264.7 cells were cultured in 24-well plates and infected with APEC94, APEC94∆cysN, and cAPEC94∆cysN cells at an MOI of 50. CFU were counted at 6 h, 12 h, 24 h, and 48 h post-infection. (**c**) Biofilm formation potential of APEC94, APEC94∆cysN, and cAPEC94∆cysN in LB at 24 h. (**d**) Adhesion of the APEC94, APEC94∆cysN, and cAPEC94∆cysN strains to RAW264.7 cells. (**e**) Invasion by the APEC94, APEC94∆cysN, and cAPEC94∆cysN strains of RAW264.7 cells. Data are means and SEM (n = 3). ^ns^ *P* > 0.05, *P < 0.05, **P < 0.01 (significant difference between the indicated groups) by one-way ANOVA. All experiments were conducted in triplicate.

The APEC94*∆*cysN strain exhibited a growth rate similar to the APEC94 and cAPEC94*∆*cysN strains, indicating that loss of the *cysN* gene did not affect APEC94’s growth rate. The results showed that APEC94 strain growth was unaffected by *cysN* deletion. No significant difference (*P* > 0.05) was observed between strains (Fig. 2a). Similarly, the bacterial count in RAW 264.7 cells infected with APEC94 was noticeably higher than in cells infected with the APEC94*∆*cysN strain up to 12 h post-infection. The APEC94*∆*cysN showed a significant defect in intracellular survival at the 12 h time point. However, this defect was overcome by 24 h, suggesting that *cysN* is important for initial persistence within macrophages (Fig. 2b). Similarly, the deletion of *cysN* resulted in a significant (35%) reduction (*P* < 0.05) in biofilm formation compared to the wild-type APEC94 strain after 24 h of incubation (Fig. 2c). Likewise, the rate of adhesion of the APEC94*∆*cysN to RAW 264.7 cells (16.29%) was significantly (*P* < 0.01) lower than that of the APEC94 strain (23.36%) (Fig. 2d). The invasion rate of the APEC94*∆*cysN strain to RAW 264.7 cells (12.54%) was significantly lower than (*P* < 0.05) that of the APEC94 (17.36%) (Fig. 2e).

### Deletion of *cysN* affects APEC94 motility

**Fig 3:**
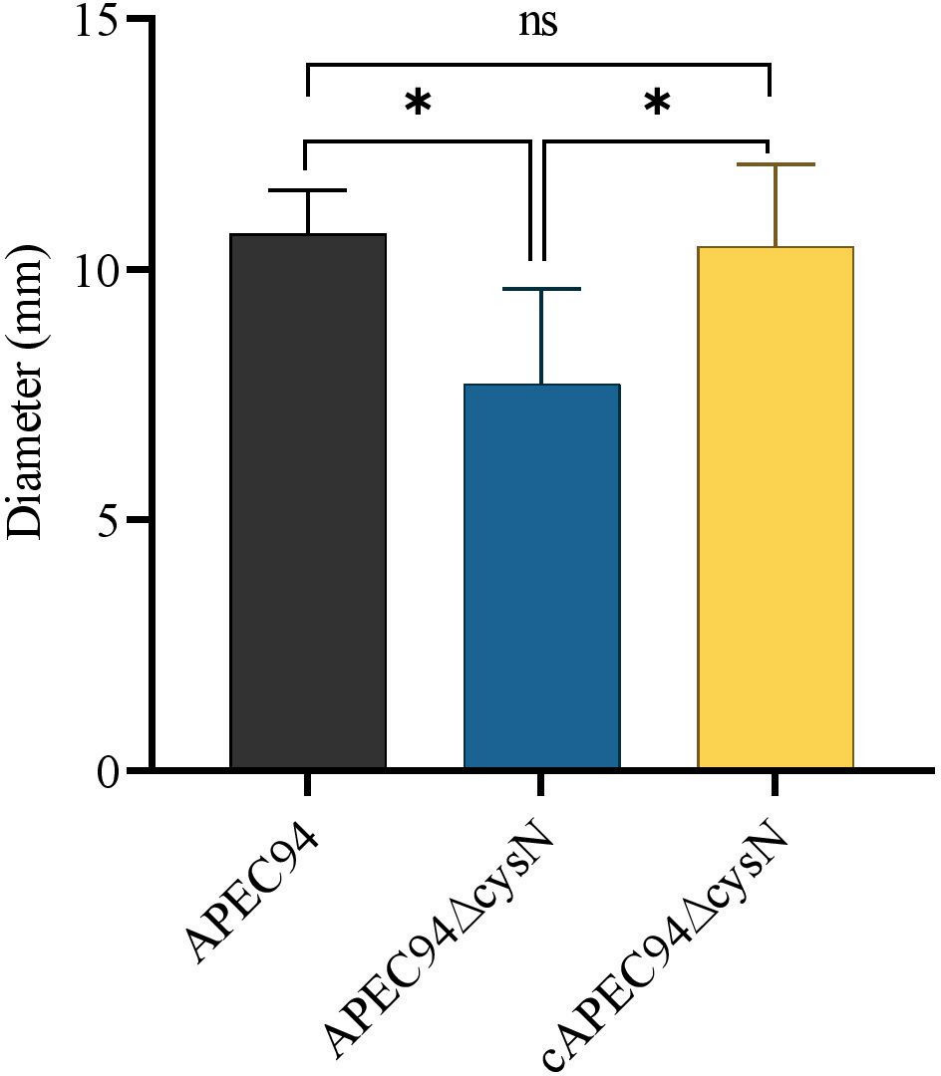
Bacterial motility. The motility diameter of the mutant strain was similar to that of the wild-type and complemented strains. Each value represents the average of three independent experiments. Significant differences were detected using one-way ANOVA (*P < 0.05, ^ns^*P* > 0.05).

The APEC94∆cysN motility significantly decreased (*P* < 0.05) compared to the APEC94 and cAPEC94∆cysN. A significant difference in the halo diameter on swarming agar plates was observed among the APEC94∆cysN, cAPEC94∆cysN, and APEC94 strains, which indicates that the deletion of *cysN* had a significant effect on the motility of APEC94 (Fig. 3).

### Antibiotic susceptibility testing for APEC94 and mutant strains

The results showed that deleting *cysN* significantly altered the antibiotic resistance of APEC94 (Table 3). APEC94 was resistant to kanamycin, fosfomycin, and clindamycin. However, it was highly susceptible to sulfamethoxazole/trimethoprim and tetracycline (Fig. S1a). In contrast, the APEC94∆cysN strain was resistant to tetracycline and sulfamethoxazole/trimethoprim but highly susceptible to clindamycin, kanamycin, and fosfomycin (Fig. S1b). Similarly, the cAPEC94∆cysN strain was susceptible to clindamycin, fosfomycin, streptomycin, and ampicillin (Fig. S1c). ATCC25592 was used as a control. All antibiotics were effective (Fig. S1d).

### Altered expression of efflux pump genes (*acrA* and *tolC*) in the APEC94ΔcysN strain

**Fig 4:**
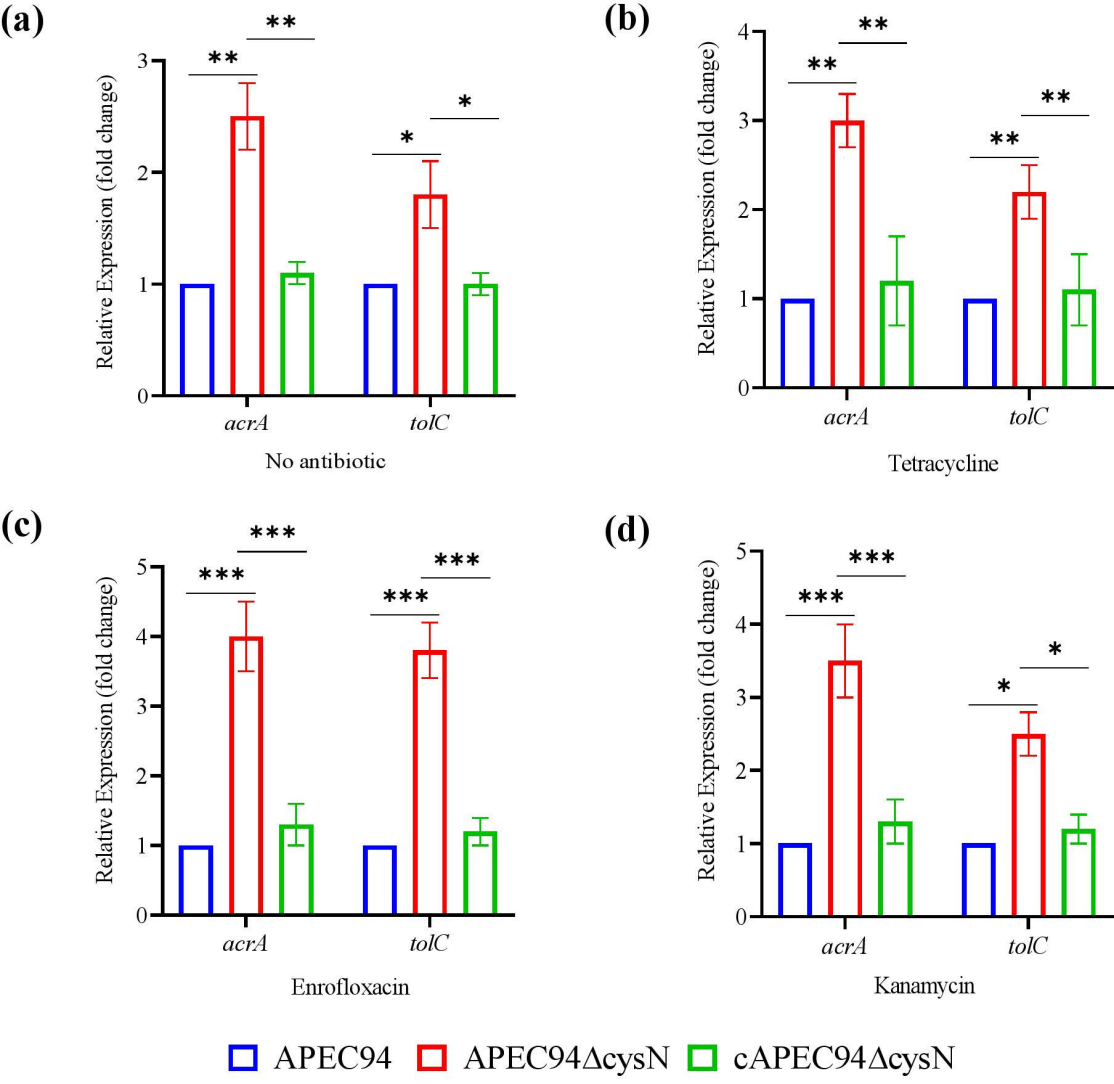
qRT-PCR analysis of efflux pump gene expression (*acrA* and *tolC*). Relative mRNA expression of efflux pump genes (*acrA* and *tolC*) in APEC94, APEC94ΔcysN, and cAPEC94ΔcysN under different antibiotic treatments. Gene expression was normalized to 16S rRNA, and the fold change was calculated using the 2^(-ΔΔCt) method. Data are presented as mean ± standard deviation (SD) of three independent experiments. Statistical significance is indicated (*P < 0.05, **P < 0.01, ***P < 0.001). (**a**) No antibiotic (control), (**b**) tetracycline (TCY-30), (**c**) enrofloxacin (ENR-5), and (**d**) kanamycin (KAN-15).

qRT-PCR analysis of *acrA* and *tolC* revealed significant changes in efflux pump genes *acrA* and *tolC* expression in the APEC94ΔcysN strain under different antibiotic treatments (Table 3). In the absence of antibiotics, the APEC94ΔcysN strain exhibited a 2.5-fold increase in *acrA* expression (*P* < 0.01) and a 1.8-fold increase in *tolC* expression (*P* < 0.05) compared to the APEC94 strain. Upon exposure to various antibiotics, the APEC94ΔcysN strain consistently showed elevated expression of *acrA* and *tolC* compared to the APEC94 strain. Under tetracycline (TCY-30 μg), *acrA* and *tolC* levels increased (*P* < 0.01) by 3.0-fold and 2.2-fold, respectively, while in the presence of kanamycin (KAN-15 μg), *acrA* and *tolC* expression reached 3.5-fold (*P* < 0.001) and 2.5-fold (*P* < 0.05), respectively. Similarly, enrofloxacin (ENR-5 μg) exposure led to the highest expression, with *acrA* and *tolC* increasing by 4.5-fold and 3.8-fold (*P* < 0.001), respectively (Fig. 4). Upregulation of *acrA* and *tolC* reflects a compensatory stress response to altered cysteine metabolism and redox imbalance. This upregulation was consistently reversed in the cAPEC94ΔcysN, similar to APEC94, supporting a regulatory association which indicates that the increased efflux pump gene expression is directly linked to the loss of *cysN*. Our findings demonstrate that *cysN* increases antibiotic susceptibility, which is linked to the upregulated expression of efflux pump genes *acrA* and *tolC* (Fig. S2).

### Deletion of *cysN* alters the MIC of APEC94

**Fig 5:**
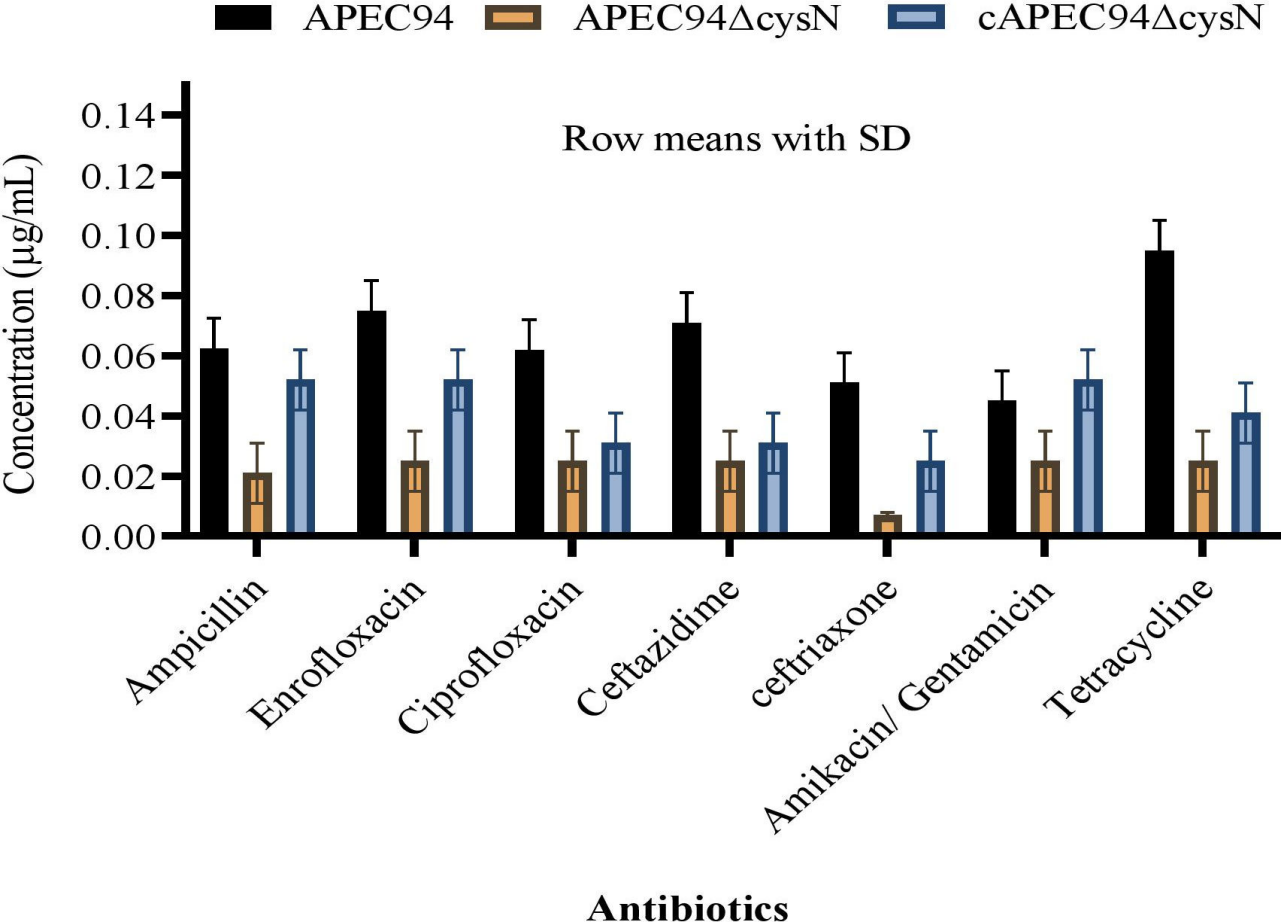
Presents the MIC results for different antibiotics against the APEC94, APEC94ΔcysN, and cAPEC94ΔcysN strains. Bar graphs represent the mean MIC values with standard deviations.

The MICs of ampicillin, enrofloxacin, ciprofloxacin, ceftazidime, ceftriaxone, amikacin, gentamicin, and tetracycline against APEC94, APEC94∆cysN, and cAPEC94∆cysN strains were determined. Our findings demonstrate that the disruption of the *cysN* gene significantly increased the MIC for ampicillin and tetracycline, indicating a reduction in susceptibility to these antibiotics. Conversely, the mutation decreased the MIC for enrofloxacin and ceftazidime, suggesting increased susceptibility. This suggests a key role for the cysteine biosynthesis pathway in modulating intrinsic antibiotic resistance in APEC (Fig. 5).

### Deletion of *cysN* attenuates serum resistance of APEC94

**Fig 6:**
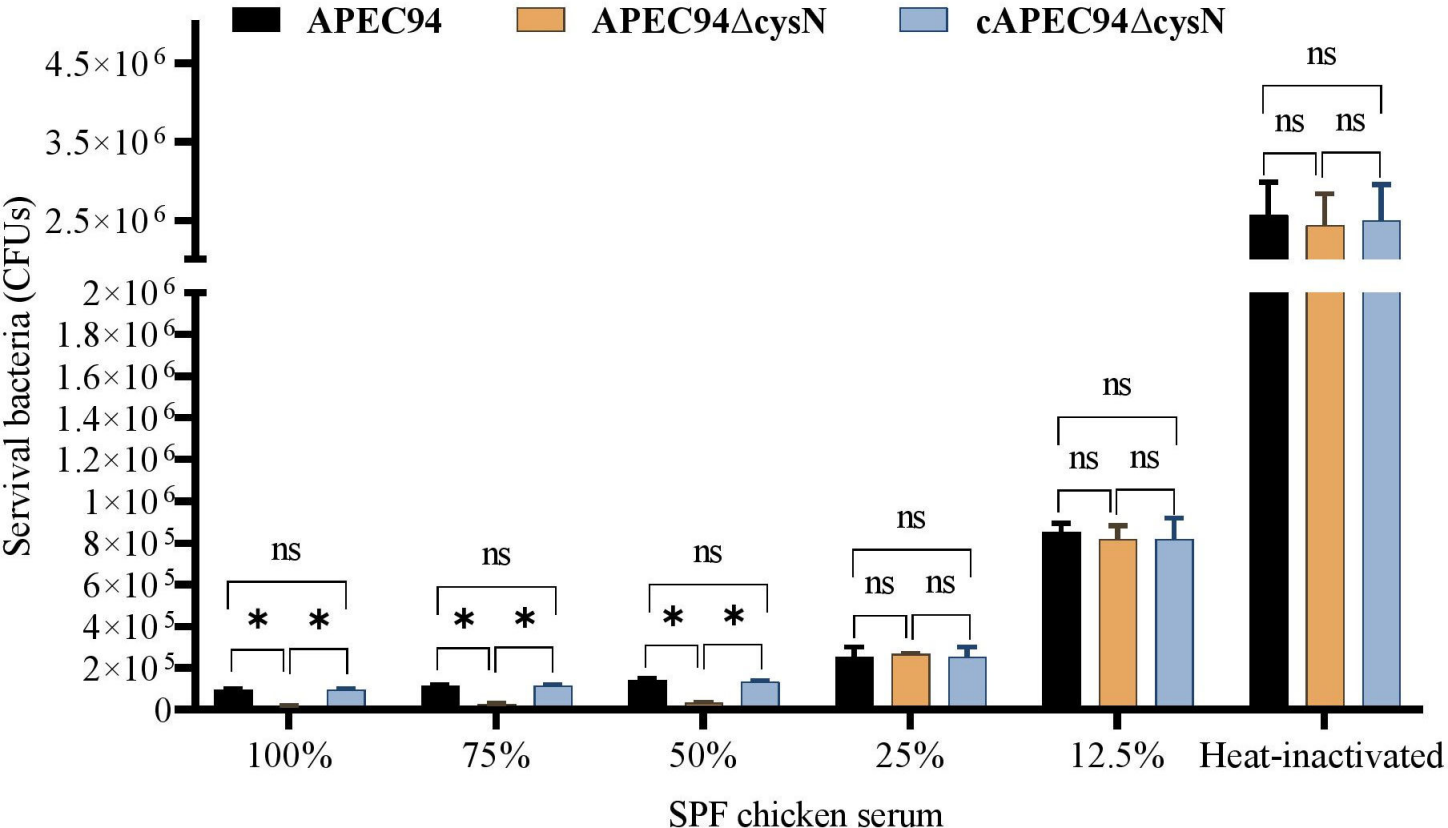
Bacterial resistance to specific-pathogen-free (SPF) chicken serum. APEC strains were incubated with different dilutions of SPF chicken serum at 37°C for 30 min, and the surviving bacteria were counted. The APEC94∆cysN strain shows decreased survival in SPF chicken serum compared with APEC94 and cAPEC94∆cysN strains. A two-way ANOVA was performed for the survival assays (*P < 0.05, ^ns^*P* > 0.05).

Resistance to bactericidal serum is essential for APEC’s systemic dissemination and survival during infection. To assess this, the effect of *cysN* on the serum resistance of APEC94∆cysN was determined. The results showed that the survival of the APEC94*∆*cysN strain was significantly reduced (*P* < 0.05) in 100%, 75%, and 50% specific-pathogen-free (SPF) chicken serum compared to APEC94 and the cAPEC94∆cysN strain. Notably, the serum resistance defects were restored in the cAPEC94∆cysN strain. In contrast, no differences (*P* > 0.05) were observed among APEC94, APEC94∆cysN, and cAPEC94∆cysN strains in heat-inactivated serum, likely due to the inactivation of functional antibacterial factors, such as complement. These findings indicate that *cysN* is required for full serum resistance in APEC94 (Fig. 6).

### *cysN* deletion downregulates flagellar and type 1 fimbria gene expression

**Fig 7:**
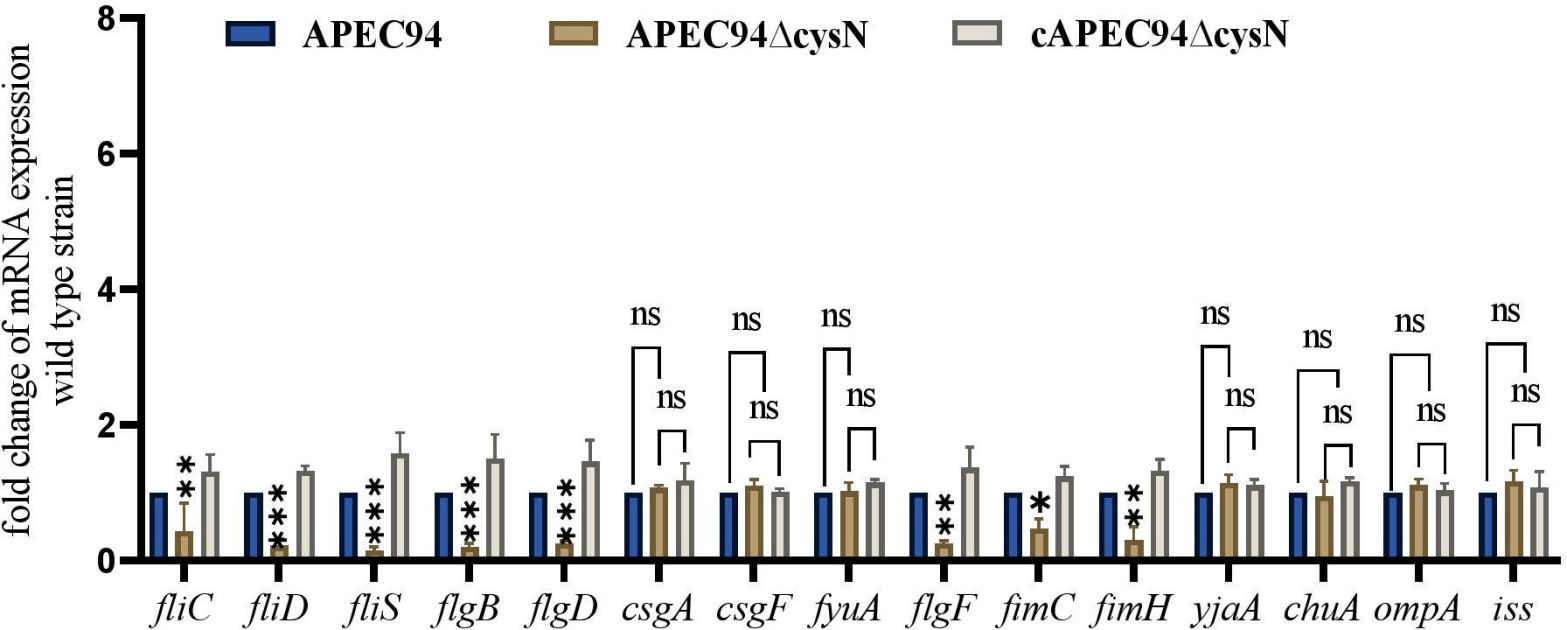
Quantitative analysis of flagellar, type 1 fimbria, and virulence gene transcription levels in APEC. qRT-PCR was performed to measure the transcription levels of flagellar, type 1 fimbria, and virulence genes associated with serum resistance in APEC94, APEC94∆cysN, and cAPEC94∆cysN strains. Transcription levels were normalized to the housekeeping gene dnaE. Expression of *iss*, *chuA*, *ompA*, *csgA*, *csgF*, *yjaA*, and *fyuA* was not significantly altered. The results are shown as relative expression ratios compared to expression in the APEC94 strain. Two-way ANOVA was conducted to determine statistical significance (^ns^*P* > 0.05, **P < 0.01, ***P < 0.001).

The transcription levels of the flagellar genes (*fliC*, *flgB*, *flgD*, *fliD*, *fliS*, and *flgF*), the type 1 fimbrial genes (*fimC*, *fimH*), and the virulence genes (*csgA*, *iss*, *csgF*, *fyuA*, *yjaA*, *chuA*, and *ompA*) in the APEC94∆cysN strain were quantified as fold changes relative to those of the APEC94. The mRNA levels of the *fliC*, *fliD*, *fliS*, *flgB, flgD*, *flgF*, *fimC*, and *fimH* genes were significantly downregulated (*P* < 0.01, *P* < 0.001) in the APEC94∆cysN compared with those in the APEC94 strain. The expression levels of these genes were restored in the cAPEC94∆cysN strain (*P* > 0.05). However, the expression levels of virulence genes, such as *csgA, csgF*, *fyuA, yjaA, chuA, ompA*, and *iss*, were not influenced (*P* > 0.05) compared to the APEC94 strain. This suggests the role of *cysN* is specific to surface structure and motility-associated virulence factors, not a global regulator of all virulence genes (Fig. 7).

### *cysN* contributes to APEC94 virulence *in vivo*

**Fig 8:**
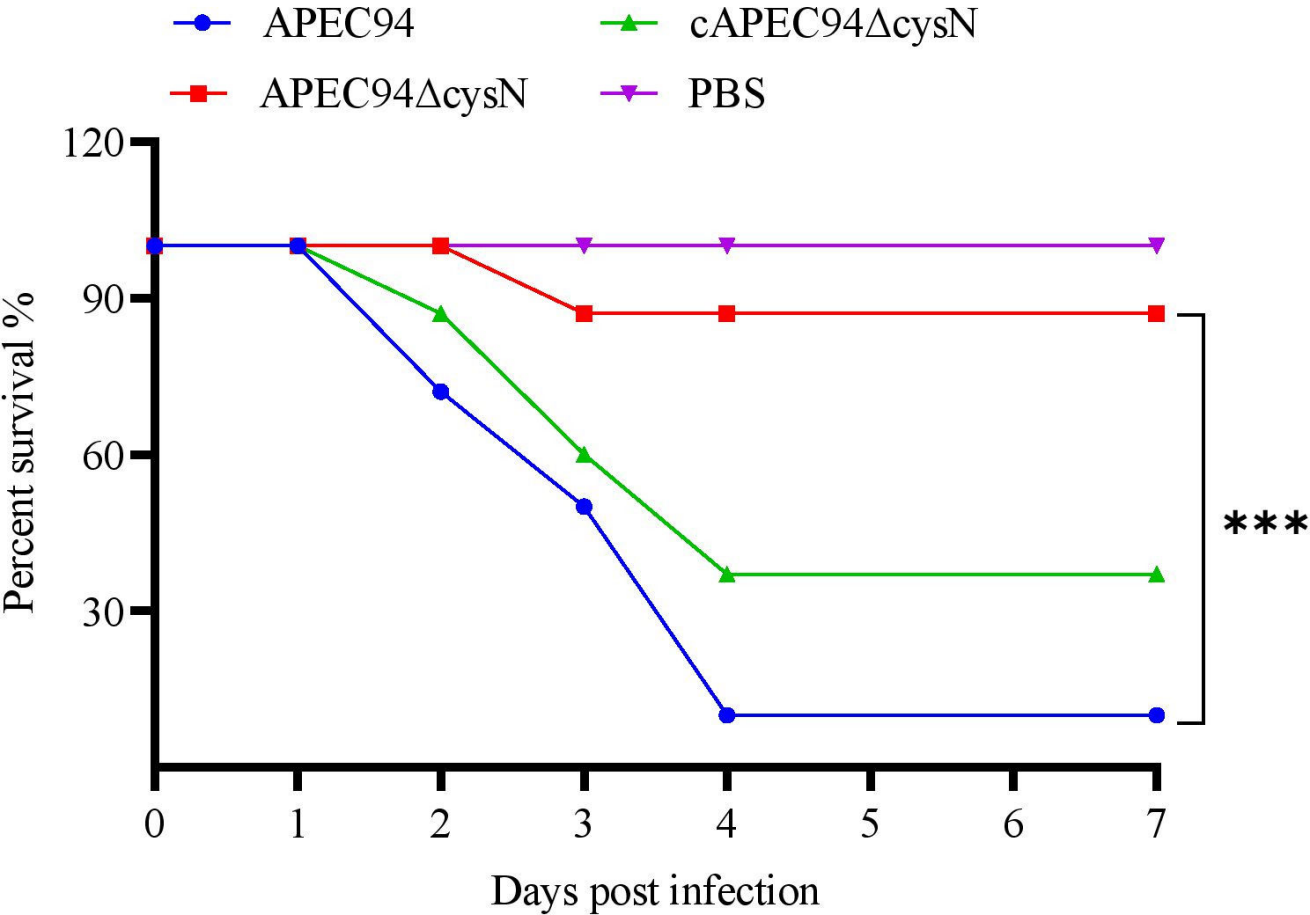
*cysN* contributes to APEC94 virulence in a murine model. Mice aged 6 weeks were injected intraperitoneally with 10^6^ CFU of APEC94, APEC94∆cysN, and cAPEC94∆cysN strains, or a negative control, PBS. Survival until 7 days post-infection, and the percentage of survival was determined. The APEC94∆cysN exhibited lower pathogenicity when compared to the APEC94 and cAPEC94∆cysN strains (***P < 0.001).

To determine the effect of *cysN* on APEC pathogenicity, mice infection models were used to investigate the virulence of APEC94, APEC94∆cysN, and cAPEC94∆cysN strains. It is worth noting that this model serves as an approximation for natural avian infection, and implications for poultry should be drawn cautiously. The survival curves show that mice infected intraperitoneally with APEC94, APEC94ΔcysN, and cAPEC94ΔcysN had mortality rates of 87.5% (7/8), 12.5% (1/8), and 62.5% (5/8), respectively (*P* < 0.001). As a result, *cysN* deletion reduced APEC virulence in mice, which could be partly restored in the complemented strain. These findings indicate that *cysN* contributes to the pathogenicity of APEC (Fig. 8).

### *cysN* contributes to APEC94 colonization *in vivo*

**Fig 9:**
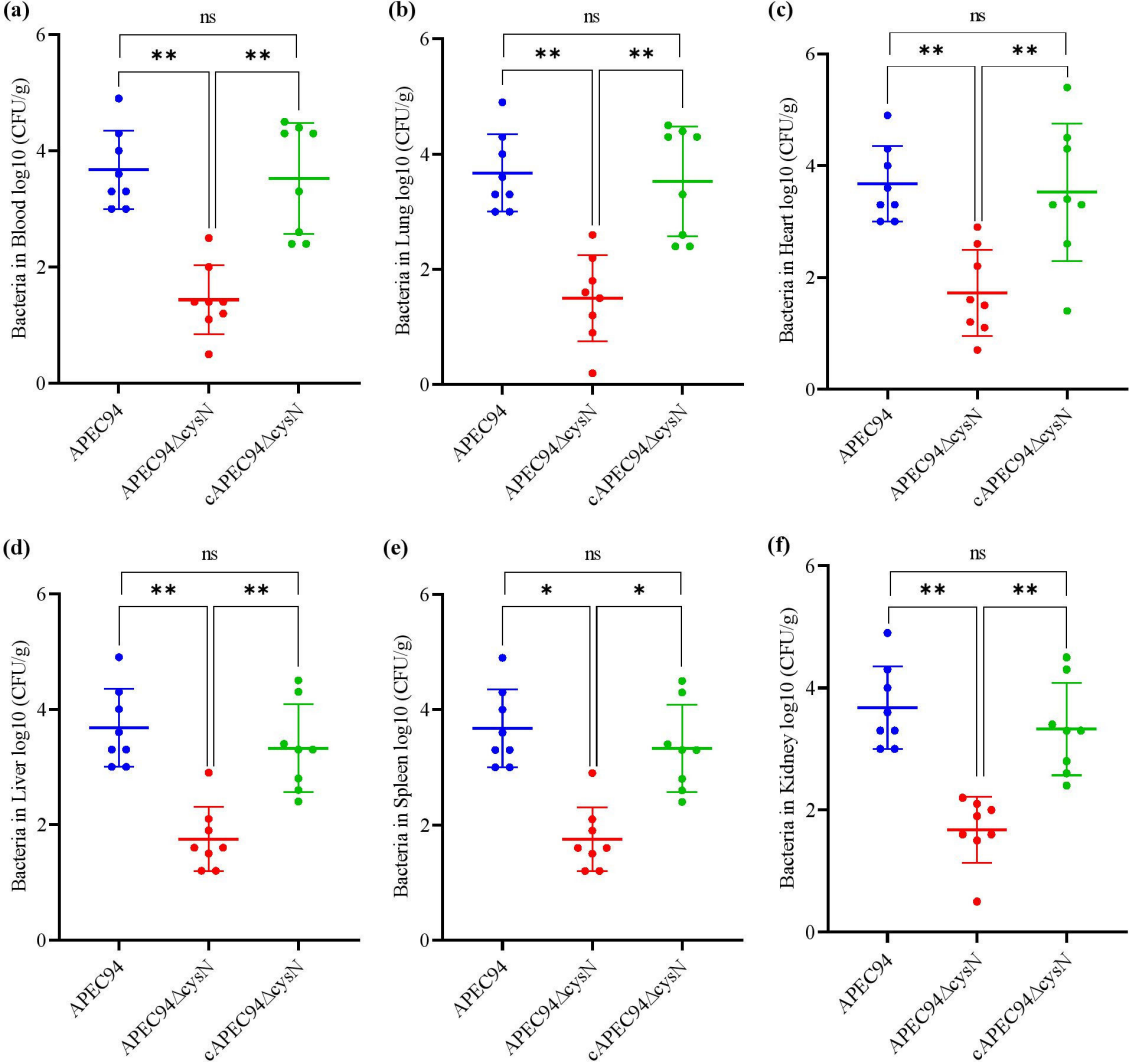
*cysN* contributes to APEC94 colonization in a murine model. APEC strains colonize and survive in mice following systemic infection. Six-week-old mice were injected intraperitoneally. Graphs indicate bacterial titers recovered from the blood (**a**), lung (**b**), heart (**c**), liver (**d**), spleen (**e**), and kidney (**f**) 24 h post-infection. The APEC94∆cysN strain lowered the bacterial burdens in the infected tissues of mice compared to the APEC94 and cAPEC94∆cysN strains. The Mann–Whitney U Test was used to assess statistical significance (**P < 0.01, *P < 0.05, ^ns^*P* > 0.05).

A systemic infection experiment was conducted in mice to assess the effect of *cysN* on APEC colonization *in vivo*. Bacterial loads in the mice tissues were tested 24 h after infection. The bacterial tissue loads in the blood, lung, heart, liver, spleen, and kidney of the mutant APEC94∆cysN strain showed a significant reduction (*P* < 0.01, *P* < 0.05) compared to the APEC94 strain. The colonization of bacteria was restored in complemented cAPEC94∆cysN strains (*P* > 0.05). These findings suggest that the gene *cysN* is involved in colonization during systemic infection *in vivo* (Fig. 9).

### Determination of inflammatory cytokine transcription in APEC-infected avian tissues

**Fig 10:**
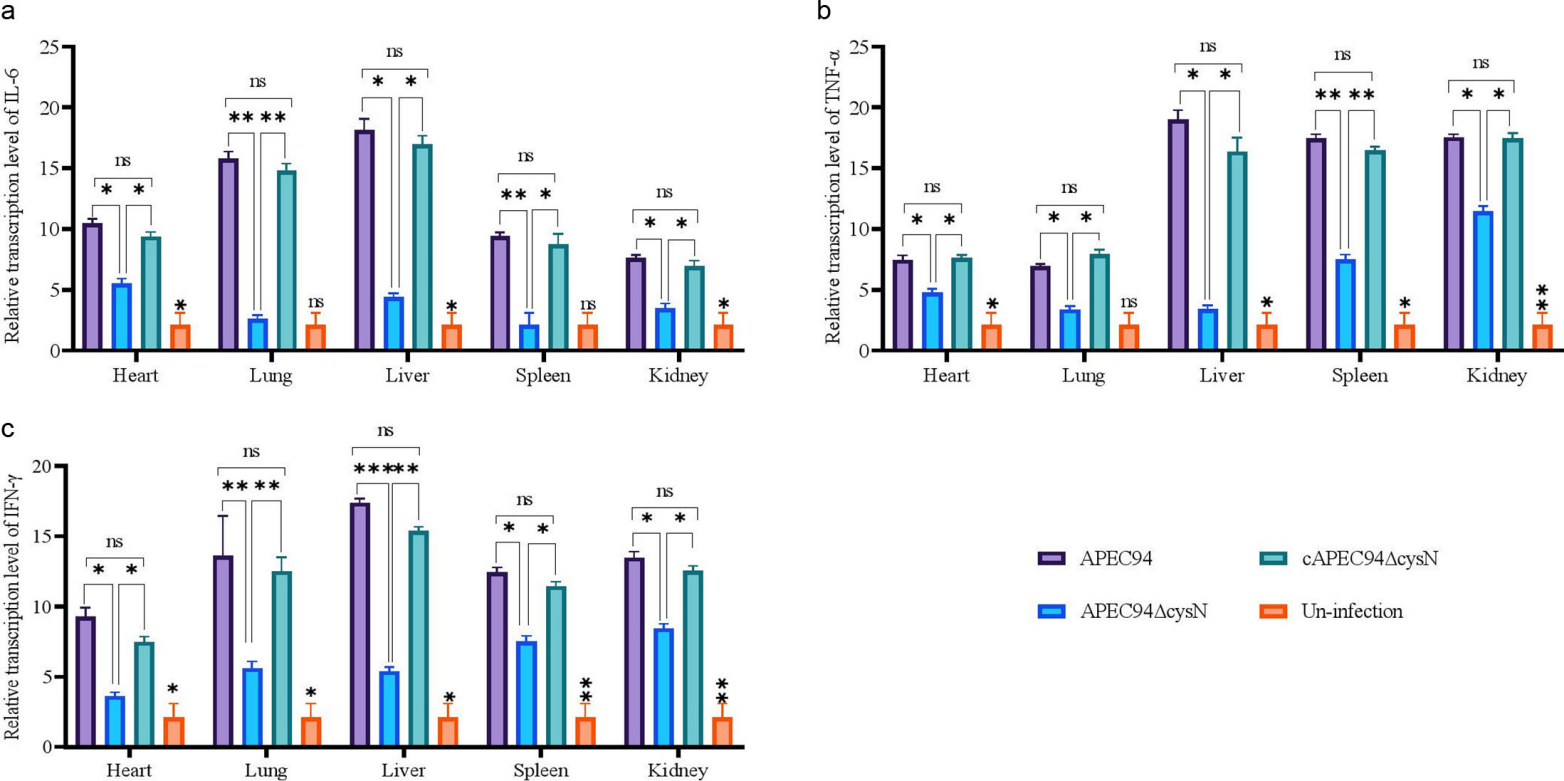
APEC94∆cysN infection attenuates the pro-inflammatory cytokine response in a murine model. Tissues were collected from mice infected with APEC strains at 24 h post-inoculation. The transcription levels of key pro-inflammatory cytokines were analyzed by qRT-PCR. APEC94 infection led to significantly upregulated transcription of (**a**) IL-6, (**b**) TNF-α, and (**c**) IFN-ƴ compared to uninfected tissues. This inflammatory response was significantly decreased in tissues infected with APEC94∆cysN. Gene expression was normalized to *GAPDH*. Statistical significance was assessed by two-way ANOVA (***P < 0.001, **P < 0.01, *P < 0.05, ^ns^*P* > 0.05) (Fig. S3).

The pathogen infection could activate macrophages and promote the transcription of inflammatory cytokines (24). The transcription of inflammatory cytokines was assessed in mice infected with APEC94, APEC94∆cysN, and cAPEC94∆cysN strains in the heart, lung, liver, spleen, and kidney 24 h post-infection by qRT-PCR. Figure 10 shows that APEC-infected tissues had considerably higher levels of transcription of IL-6, TNF-α, and INF-ƴ compared to uninfected tissues. Compared to that treated with APEC94, the transcription of these inflammatory cytokines was significantly downregulated in APEC94ΔcysN-infected tissues (*P* < 0.001, *P* < 0.01, *P* < 0.05) at 24 h post-infection. Moreover, the transcription of these inflammatory cytokines in cAPEC94ΔcysN-infected tissues was restored (*P* > 0.05). The transcription levels of IL-2, IL-4, and IL-10 are shown in Fig. S3. Overall, the data indicate that *cysN* deletion led to a significant reduction in pro-inflammatory cytokines, such as IL-2 and IL-6, in most tissues. At the same time, anti-inflammatory IL-4 was often elevated, suggesting *cysN’s* role in provoking a robust inflammatory response.

### Cytokine profiles in serum and tissues post-infection

**Fig 11:**
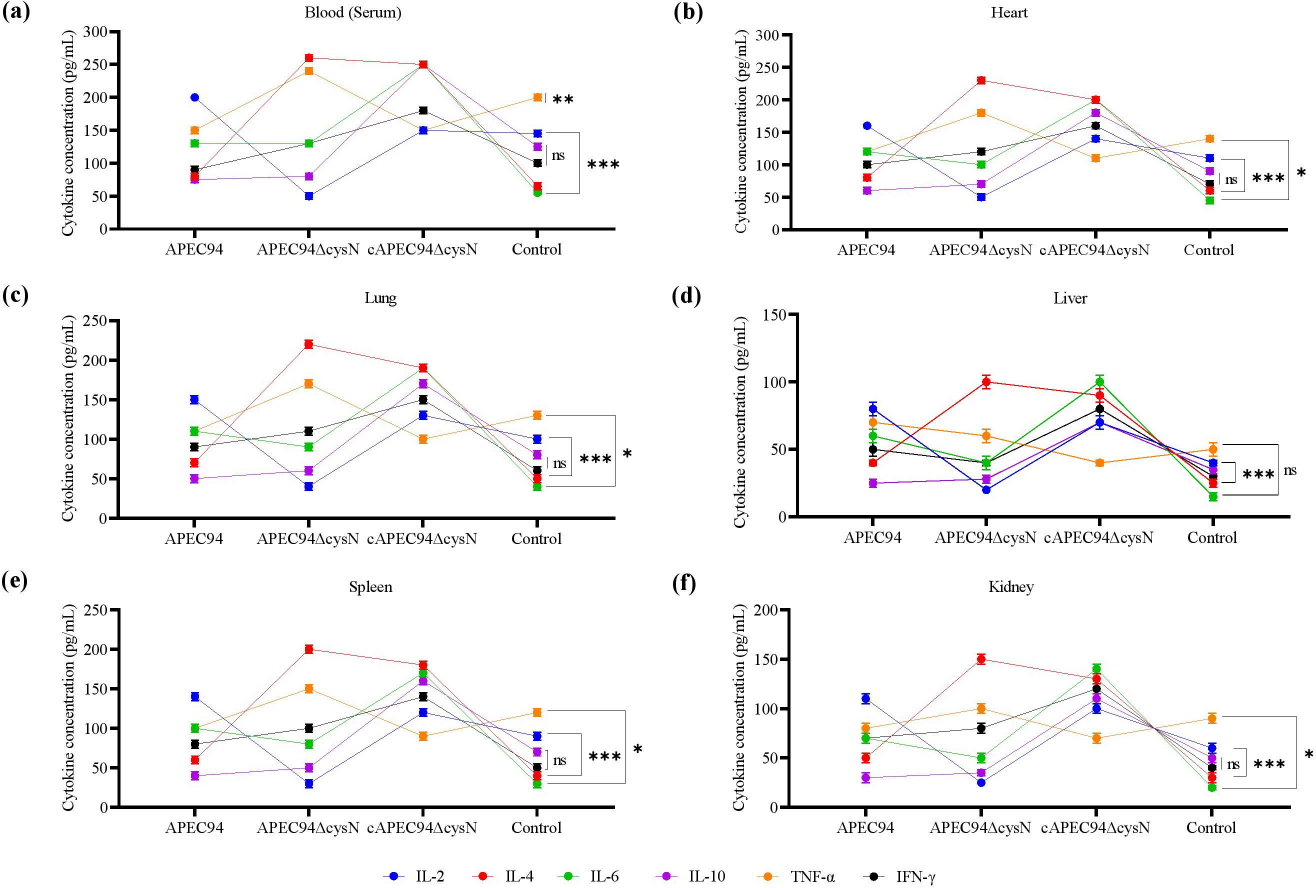
Quantification of IL-2, IL-4, IL-6, IL-10, TNF-α, and IFN-γ cytokine levels in (**a**) serum, (**b**) heart, (**c**) lung, (**d**) liver, (**e**) spleen, and (**f**) kidney of mice infected with APEC94, APEC94ΔcysN, and cAPEC94ΔcysN strains. Cytokine levels were measured using the MILLIPLEX Mouse Cytokine/Chemokine Magnetic Bead Panel–Premixed 32 Plex. Data represent mean ± SD from three independent experiments. Statistical significance was assessed by one-way ANOVA followed by Tukey's post-hoc test. (^ns^*P* > 0.05, *P < 0.05, **P < 0.01, ***P < 0.001).

Cytokine profiling indicated that mice infected with APEC94ΔcysN showed diminished levels of the inflammatory cytokine IL-2 (*P* < 0.001), elevated levels of IL-4 (*P* < 0.001) and TNF-α (*P* < 0.01) in serum when compared to those infected with the APEC94 strain (Fig. 11a). In both heart and lung, a similar trend was observed, where the APEC94ΔcysN-infected group exhibited reduced cytokine level IL-6 (*P* < 0.05), which was restored upon complementation with the cAPEC94ΔcysN strain (Fig. 11b and c). These findings highlight the essential function of *cysN* in regulating pulmonary immune responses. In the liver, the level of IL-2 cytokine is significantly reduced (*P* < 0.001). Elevated cytokine levels in IL-4 (*P* < 0.001) were observed, alongside the partial recovery in the cAPEC94ΔcysN group (Fig. 11d). Furthermore, the spleen showing decreased levels of IL-2 (*P* < 0.001) and IL-6 (*P* < 0.05) exhibited increased levels of IL-4 (*P* < 0.001) and TNF-α (*P* < 0.05) in the APEC94ΔcysN group, suggesting systemic immunosuppression due to deletion of *cysN* (Fig. 11e). Similarly, the kidney was characterized by reduced expression of IL-2 (*P* < 0.001) and IL-6 (*P* < 0.05), along with elevated levels of IL-4 (*P* < 0.001) and TNF-α (*P* < 0.05) in the APEC94ΔcysN group. This pattern suggests a possible shift from Th1-type (IL-2–driven) responses to Th2-type (IL-4–driven) responses in APEC94∆cysN-infected mice. There was no significant effect observed in the cytokine levels of IL-10 and IFN-γ (*P* > 0.05) in APEC94ΔcysN-infected serum and tissues (Fig. 11f).

## DISCUSSION

In this study, we hypothesized that the QS regulator LsrR regulates *cysN* and that this pathway is a critical determinant of APEC virulence. Our findings confirm this hypothesis: first, by demonstrating that LsrR binds specifically to a 30 bp motif in the *cysN* promoter, and second, by establishing through transcriptional analysis that LsrR acts as a direct positive regulator of *cysN*. This regulatory connection explains why the deletion of *cysN* significantly impairs APEC’s physiological characteristics, including virulence, motility, antibiotic resistance, serum survival, and host cell damage both *in vitro* and *in vivo*. Mechanistically, LsrR was shown to bind specifically to a 30 bp motif located between −289 and −319 upstream of the *cysN* gene, as confirmed by bacterial two-hybrid analysis, EMSA, and DNase I footprinting assays. Mutational analysis of this region identified conserved TCCG and GGAAAA motifs essential for LsrR binding, further supporting a direct and sequence-specific regulatory interaction.

LsrR is well-recognized for modulating QS in *E. coli*, yet its comprehensive transcriptional targets and regulatory mechanisms remain largely unexplored. In this study, we successfully expressed and purified LsrR and demonstrated that it specifically binds to the promoter region of *cysN* through a series of interaction and binding assays (13, 25). The function of *cysN* in the pathogenesis of APEC was evaluated by constructing a knockout APEC94ΔcysN strain and its complemented derivative (26). Although *cysN* deletion had no impact on bacterial growth *in vitro*, it significantly reduced key virulence traits, including adhesion, invasion, and biofilm formation in RAW 264.7 macrophage cells (27). These phenotypes were largely restored in the complemented strain, confirming the contribution of *cysN* to these virulence-associated functions. Our findings demonstrate that the deletion of *cysN* modifies the biological properties of APEC94 bacteria, particularly those related to host interaction and persistence. This finding is consistent with previous studies in other pathogens, such as *Pseudomonas aeruginosa*, where dispersed cells derived from biofilms exhibit altered expression of virulence factors that enhance host colonization and dissemination (28).

Motility plays a crucial role in host colonization and systemic spread of pathogens. The absence of *cysN* led to a marked decrease in swarming motility, which was associated with the downregulation of flagellar genes, including *fliC*, *fliD*, *fliS*, *flgB*, *flgD*, *flgF*, *fimC*, and *fimH,* as assessed by qRT-PCR (29). These results indicate that *cysN* has a positive influence on the expression of flagellar operons and is essential for motility-associated behavior in APEC (30).

One of the most intriguing findings is the altered antibiotic susceptibility profile of the APEC94ΔcysN mutant, which was associated with a significant upregulation of the *acrA* and *tolC* efflux pump genes. The APEC94ΔcysN strain revealed enhanced resistance to tetracycline and sulfamethoxazole/trimethoprim, while showing increased sensitivity to aminoglycosides and fosfomycin compared to the APEC94 strain (31). This shift in resistance patterns was associated with a significant upregulation of the efflux pump genes *acrA* and *tolC* in the APEC94ΔcysN strain (32). The effect was especially pronounced under conditions of antibiotic stress, with the expression of these genes increasing up to 4.5-fold (33). The restoration of efflux gene expression in the complemented strain highlights the regulatory influence of *cysN* on efflux-mediated drug resistance, indicating that *cysN* alters efflux pump gene expression, possibly via metabolic stress and QS-mediated pathways (34). Previous assessments of *E. coli’s* antibiotic resistance profile indicated a resistance rate of approximately 70% to streptomycin, sulfisoxazole, and tetracycline, along with a noted decrease in susceptibility to ampicillin, kanamycin, sulfisoxazole, streptomycin, tetracycline, and ticarcillin (35). Resistance to ceftiofur, ceftriaxone, cephalothin, ampicillin, and amoxicillin-clavulanic acid has also been documented (36, 37). The MIC for APEC94 and its mutant strains was evaluated, yielding significant results in accordance with the EUCAST guidelines (14). Additionally, prior research indicated that sub-MIC levels of ceftazidime could hinder biofilm formation in *E. coli* (38) by increasing extracellular indole concentrations and downregulating the *cysN* and *ibpA* genes (39).

The ability to resist host serum is a hallmark of systemic infection caused by APEC (40). The survival of APEC94ΔcysN was notably diminished in chicken serum when compared to both wild-type and complemented strains (19). These differences were not observed in heat-inactivated serum, confirming the involvement of functional complement in bacterial killing (41). These findings suggest that *cysN* contributes to APEC’s complement resistance, thereby supporting its systemic pathogenic potential (42).

Notably, while *cysN* has been shown to influence the expression of fimbrial genes (*fimC*, *fimH*), which correlates with reduced adhesion (40) and invasion, it did not seem to impact the expression of other significant virulence genes such as *iss*, *chuA*, *ompA*, and *fyuA* (19, 41, 42). This indicates that the regulatory role of *cysN* is likely confined to a specific subset of virulence pathways, particularly those associated with surface structures and motility, rather than serving as a general regulator of virulence gene expression (43). Although specific inhibitors for *cysN* have yet to be discovered, the pathways involved in sulfur assimilation and cysteine biosynthesis represent promising avenues for antimicrobial development. The inhibition of genes, such as *cysE*, *cysK*, and APS reductase (*cysH*), disrupts the redox balance and diminishes virulence in pathogens, such as *Mycobacterium tuberculosis*, suggesting that targeting sulfur metabolism may be a viable strategy for combating bacterial infections (7, 8).

Additionally, pathogenic bacteria often manipulate host immune and inflammatory responses to enhance their survival and proliferation (24, 44). Cytokines are crucial regulators of these responses, and their dysregulation can profoundly alter the dynamics between host and pathogen. In this study, deletion of *cysN* led to a distinct and tissue-specific cytokine profile. Mice infected with the APEC94ΔcysN mutant exhibited a consistent pattern of diminished IL-2 and IL-6 alongside elevated IL-4 and TNF-α across multiple tissues. Mechanistically, this profile suggests that the loss of *cysN* impairs the robust pro-inflammatory response typically required for effective bacterial clearance. The reduction in key T-cell and macrophage activators (IL-2, IL-6), coupled with an increase in the Th2-associated cytokine IL-4, indicates a skewed immune response away from protective Th1/Th17 immunity and toward a less effective Th2 polarization. This immune imbalance likely reduces the efficiency of pathogen clearance, as critical bactericidal mechanisms are not fully activated. Furthermore, the concurrent elevation of TNF-α suggests an altered and potentially dysregulated inflammatory pathology, which may contribute to tissue damage without effectively containing the infection. This altered immune dynamic can be directly linked to the attenuated virulence of the APEC94ΔcysN mutant. The significantly reduced bacterial load in host tissues (Fig. 9) fails to provide the strong stimulus necessary to drive a robust, protective Th1-polarized response. Consequently, the weak inflammatory environment may default toward a Th2 state, while the elevated TNF-α likely represents a dysregulated, non-productive inflammatory signal. These findings collectively demonstrate that *cysN* is essential for orchestrating the pro-inflammatory immune environment characteristic of a successful APEC infection. This state of impaired immune modulation (45, 46) ultimately compromises the host’s ability to mount a coordinated defense, reflecting a significant reduction in the strain’s overall virulence.

Notably, the anti-inflammatory cytokine IL-10 and IFN-γ showed no significant variation in serum and tissues, suggesting that *cysN* deletion triggers heterogeneous immune responses. Complementation with the cAPEC94ΔcysN strain largely reinstated normal cytokine expression patterns, thereby confirming the regulatory function of *cysN* in modulating host immunity. These results bolster the hypothesis that *cysN* is not solely involved in sulfur metabolism but also plays a multifaceted role in regulating virulence-associated immune responses through its influence on QS pathways. This immune-modulatory effect corresponds with the observed reductions in bacterial virulence, motility, and serum resistance, underscoring *cysN* as a potential molecular target for anti-APEC interventions. Our research offers novel mechanistic insights into how *cysN* affects host immune signaling and cytokine dynamics during APEC infection (47, 48).

In summary, our work defines the LsrR-*cysN* axis as a novel regulatory node controlling multiple key virulence phenotypes in APEC. This pathway represents a promising target for developing anti-virulence strategies to combat APEC infections, particularly in the face of rising antibiotic resistance.

### Limitation

This study has several limitations. It is a preliminary investigation constrained by ethical and logistical factors, and it utilized a well-established murine model rather than the avian infection model. Therefore, the findings and their implications for pathogenesis are based on a murine model and should be extrapolated to the natural avian host with caution. Subsequent research should include avian models to validate our findings. While this study focused on significant phenotypic and gene expression analyses, future work employing global proteomic or transcriptomic profiling will be crucial to elucidate the comprehensive regulatory networks governed by *cysN*, clarify the nature of its interaction with LsrR (direct or indirect), and uncover novel mediators of APEC virulence and resistance.

### Conclusion

Our work establishes the LsrR-cysN axis as a critical link between bacterial signaling and core physiological processes. This reveals a vulnerable point in APEC’s adaptability, suggesting that anti-virulence approaches targeting this regulatory hub could effectively curb infection without applying direct selective pressure for resistance.

## MATERIALS AND METHODS

### Protein expression and purification

A colony from LB agar plates containing BL21 bacteria was picked, reconstituted in LB broth, and incubated at 37°C for 12 h. Then, a single colony from the expression plasmid pET-28a on LB agar plates with ampicillin (1 µL ampicillin in 1 mL LB broth) was inoculated in LB ampicillin-resistant broth and incubated at 37°C for 3 to 4 h. The OD value was measured hourly until it reached 0.4 to 0.9, after which isopropylthiogalactoside (0.1 mM IPTG; 0.5 µL in 1 mL culture) was added, and the mixture was incubated at 18°C for 16 h. The sample was centrifuged at 6,000 × *g* for 30 min at 4°C. The supernatant was discarded, and the bacterial pellet was rinsed with phosphate-buffered saline (PBS). Then, 40 mL of PBS was added to the pellet, which was gently mixed, centrifuged, and washed three to four times, followed by ultrasonication and centrifugation at 6,000 × *g* for 10 min at 4°C. The supernatant was collected and sterilized using a 0.45 µm filter. The supernatant containing protein was purified using a GST resin tube. Then, 1× GST was flowed through the special GST-tag resin tube at rates of 500 µL, 600 µL, 800 µL, and 1 mL, and the protein samples were collected in new sterile Eppendorf tubes. Next, 12.5 µL loading dye was added to the 50 µL purified protein sample, which was boiled for 10 min. The SDS-PAGE model was properly arranged, and the sample was run. A 5 µL protein marker and a 10 µL protein sample were loaded into the polyacrylamide gel, and PAGE electrophoresis was performed at 100 V for 90 min. After PAGE completion, dyeing and decolorizing with Coomassie and decolorizer solution were performed to achieve clear bands, and the correct LsrR protein size was analyzed.

### Construction of mutant and complemented strains

The mutant and complemented strains were constructed using the λ red recombinase system (49) with additional modifications and the required primers. All primers used in this study are listed in Table 1. The upstream and downstream regions of the *cysN* gene were amplified through PCR utilizing the cysN-UF/cysN-UR and cysN-DF/cysN-DR primer sets. The ampicillin resistance gene (Amp) was also amplified from the pTarget plasmid using the pTarget-F/pTarget-R primers, resulting in the recombinant plasmid zerobank-up-Amp-down. Electroporation was employed to introduce the zerobank-up-Amp-down plasmid into DH5α competent cells, using 200 Ω, 25 µF, and 2.1 kV settings. Following a 24-hour incubation period, mutant colonies exhibiting a partial deletion of *cysN* were identified through PCR amplification with the cysN-IF/cysN-IR primer pair. Subsequently, the mutant bacterial strain was transformed with the pTarget plasmid to excise the Amp cassette, yielding an ampicillin-sensitive mutant strain. The *cysN* gene (containing the promoter) was amplified from the APEC94 using the cysN-F/cysN-R primer pair. The resulting PCR product was then cloned into the pSTV28 vector at the EcoRI and SalI restriction sites, leading to the construction of the complemented plasmid pSTV28-cysN. Finally, pSTV28-cysN was introduced into APEC94ΔcysN to generate the complemented strain cAPEC94ΔcysN. A vector-only pSTV28 control strain was also constructed and tested in preliminary assays; no significant phenotypic changes were observed compared with the APEC94.

**TABLE 1:** Oligonucleotide primers used in this study

Primer	3′−5′ sequence	Plasmid/gene	Size (bp)
*cysN* promoter Cyc 5.5 F	AATCATCGAAGAGATGCTGGTTTCCACCACCAGTGAACGTCAGGGCCGCG	*cysN*	50
cysN-F	AATCATCGAAGAGATGCTGGTTTCCACCACCAGTGAACGTCAGGGCCGCG	*cysN*	50
cysN-R	TTGTGCAAGTGCGGTGTTCATCTTAAAAATACCCCTGACGTTTTTTCAGC	*cysN*	50
cysN-IF cysN-IR	TAGGTATAATACTAGTAGCAACTTAATGACATCGGAGTTTTAGAGCTAGAAATAGC TTTTTTTCACCAAAAAAAGCACCGACTCGGT	*cysN*	700 bp
cysN-UF cysN-UR	GCTTTTTTTGCTGACCTGTGGTAGCGTCGATG ATGGCAACATACTGGCGCATCGACATGTGA	*cysN*	305
CysN-DF CysN-DR	AGCTTTCCAGTCTGCACTGGAAGGCGACAA TAATAGATCTAAGCTTCCTTTGCCGGAGAAGCGATC	*cysN*	309
D4-p-cysN-f D4-p-cysN-r	GGCTGGAAAATATCGACATT TTCCGCAGCGAGATATAGCG	*cysN*	44
M4-p-cysN-f M4-p-cysN-r	GGCTGGAAAATATCGACATT TTCCGCAGCGAGATATAGCG	*cysN*	44
M6-p-cysN-f M6-p-cysN-r	GGCAATACATCTGGCTGGAA AGCGAGATATAGCGGAACAA	*cysN*	50
D6-p-cysN-f D6-p-cysN-r	GGCAATACATCTGGCTGGAA AGCGAGATATAGCGGAACAA	*cysN*	50
Winged helix-F Winged helix-R	GACAATCAACGATTCGGCAA GCGCGACCTGTTCTTCTTCA	Winged helix	59
Sugar-binding putative-F Sugar-binding putative-R	GTTTTACTATCACGACGGGC CCTATCCCCAGTCGCCCACC	Sugar-binding putative	243
GTP binding-F GTP binding-R	CCAATGAAGGCGGCGTCGAA GGTGTCGGCGATAATAAATT	GTP binding	314
lsrR-F lsrR-R	ATGACAATCAACGATTCG TTAACTACGTAAAATCGC	*lsrR*	954
pTarget-F pTarget-R	AGCGAGGAAGCGGAAGAGCG CAAGATAGCCAGATCAATGT	pTarget	800
Pstv28-F Pstv28-R	GTAAAACGACGGCCAGT CAGGAAACAGCTATGAC	Pstv28	375
pCas-F pCas-R	GTAACATCAGAGATTTTGAGACAC GTCCACATCACTATTATC	pCas	1000
pUT18C-F pUT18C-R	GGGGAAAAGCCTGTTCGACG GACGTACACAGTCTCCAAAA	pUT18C	375
pKT25-F pKT25-R	GCCGTGGCGAAGGAAAAAA GACGTACACAGTCTCCAAAA	pKT25	375
lsrR-XbaI-pUT18c-F lsrR-ECORI-pUT18c-R	CCACTGCAGGTCGACTCTAGACATGACAATCAACGATTCGGCA CTTAGTTATATCGATGAATTCTTAACTACGTAAAATCGCCGCTG	*lsrR*/ pUT18c	970 bp
dnaE RT-F dnaE RT-R	ATGTCGGAGGCGTAAGGCT TCCAGGGCGTCAGTAAACAA	*dnaE*	181 bp
fliC RT-F fliC RT-R	ATGGCACAAGTCATTAATACCCAAC CTAACCCTGCAGCAGAGACA	*fliC*	132 bp
fliD RT-F fliD RT-R	TCGCGATGCCATTAACAACG TATCGTTAGCGGTGATGGACAG	*fliD*	*1447* bp
fliS RT-F fliS RT-R	ACTGCGGGTGAGTCTTGATG CGTTGCGTAAATTGGCTTGC	*fliS*	126 bp
flgB RT-F flgB RT-R	GCAGCAAACATCGCCAATGCC GTTGCATCCCGTCCACGTT	*flgB*	101 bp
flgD RT-F flgD RT-R	GCACAAATCAGCACGGTCAGCG ATCATCACGCCGTGACCGATCA	*flgD*	122 bp
csgA RT-F csgA RT-R	GCAATCGTATTCTCCGGTAG GATGAGCGGTCGCGTTGTTA	*csgA*	418 bp
iss RT-F iss RT-F	CCGACAGCAGTAACACCAAAGG TTCTGCACCGCCAACAAATT	*iss*	105 bp
csgF RT-F csgF RT-R	CCTCATATCAACGGCGTTTTT CCCCGGCCTGAACTTCATAG	*csgF*	*138* bp
fyuA RT-F fyuA RT-R	TTGGCGACCAGGGTAAGAGC AGACCCGCAGTAGGCACGAT	*fyuA*	145 bp
flgF RT-F flgF RT-R	ACGTCGCGTTGCAGCAGGAT TATCACCGGATGCCCCTGAA	*flgF*	128 bp
fimC RT-F fimC RT-R	CGAAAGCGCGGTTAAATTGC CCATTACGCCCGTCATTTTGG	*fimC*	127 bp
fimH RT-F fimH RT-R	TGCAGAACGGATAAGCCGTGG GCAGTCACCTGCCCTCCGGTA	*fimH*	506 bp
YjaA RT-F YjaA RT-R	TGAAGTGTCAGGAGACGCTG ATGGAGAATGCGTTCCTCAAC	*YjaA*	211 bp
chuA RT-F chuA RT-R	GACGAACCAACGGTCAGGAT TGCCGCCAGTACCAAAGACA	*chuA*	279 bp
ompA RT-F ompA RT-R	GCTGAGCCTGGGTGTTTCCT TCCAGAGCAGCCTGACCTTC	*ompA*	171 bp
acrA RT-F acrA RT-R	5′-CGAGATGGCAGACAATCGTC-3′ 5′-CGGCGATTGTGATGATGAAG-3′	*acrA*	150 bp
tolC RT-F tolC RT-R	5′-GGTTGTCAACGGCAGTAAGC-3′ 5′-CGTTCGTGAACGATGGAGAC-3′	*tolC*	120 bp
16S rRNA RT-F 16S rRNA RT-R	5′-GCAAGCGTTATCCGGAATTG-3′ 5′-TCTACGCATTTCACCGCTAC-3′	16S rRNA	150 bp

Similarly, for the construction of the APEC94ΔlsrR strain, a linear DNA fragment containing an FRT-flanked kanamycin resistance (Kan-R) cassette was generated by overlap extension PCR. This fragment was designed with 50-nucleotide homology arms corresponding to the regions immediately upstream and downstream of the *lsrR* coding sequence. The PCR product was electroporated into APEC94 cells harboring the pKD46 plasmid, which had been induced with 10 mM L-arabinose to express the λ red recombinase. Recombinants were selected on Luria-Bertani (LB) agar plates containing kanamycin (50 µg/mL). Successful deletion of the *lsrR* gene was confirmed by PCR using verification primers that bind outside the recombination region. The Kan-R cassette was subsequently excised by introducing the FLP recombinase plasmid pCP20, resulting in a clean, in-frame deletion mutant, APEC94ΔlsrR.

### Bacterial strains, plasmid constructs, and growth conditions

**Fig 12:**
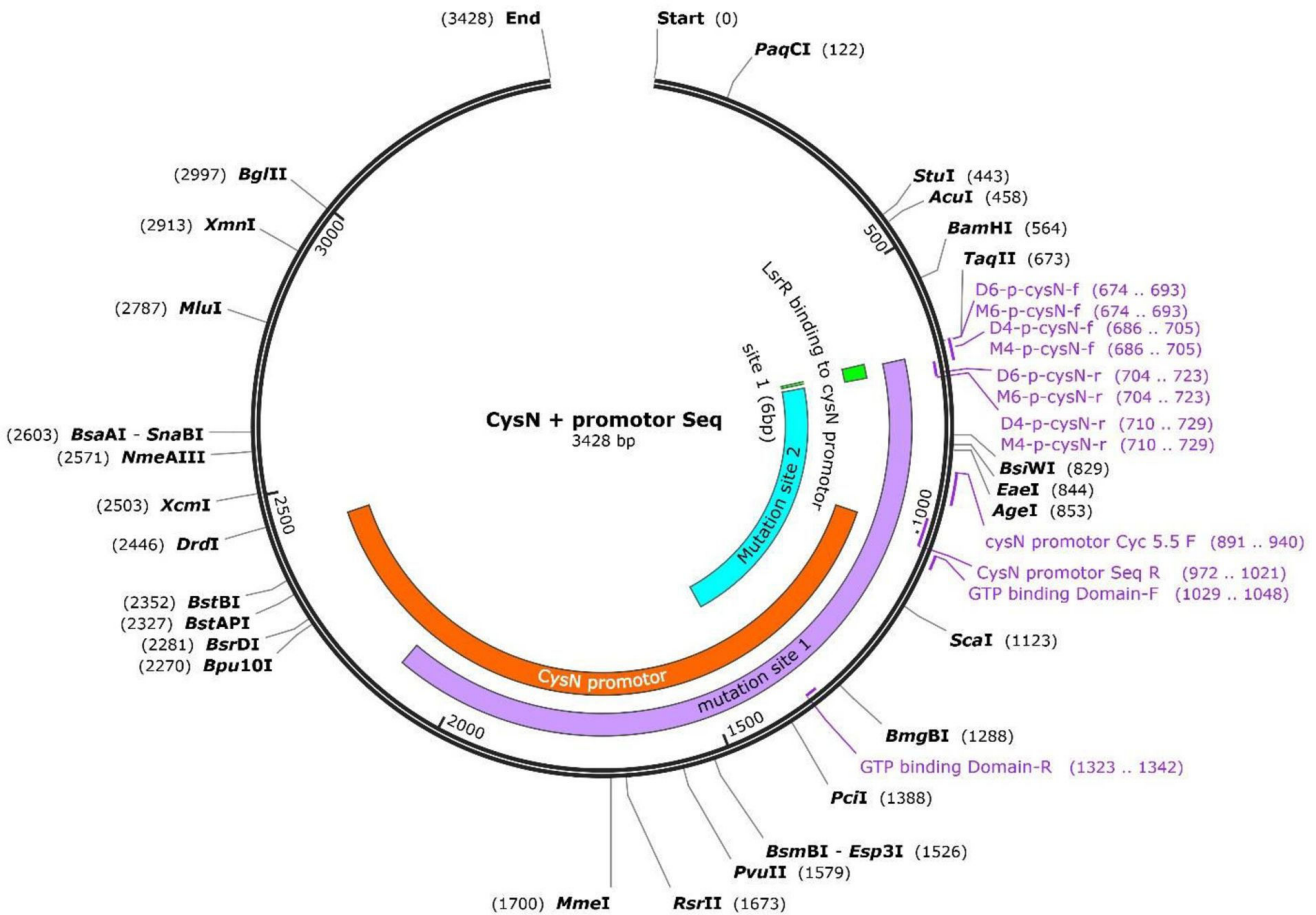
Map of a circular *cysN* promoter. It indicates the position and size of each fragment (D4-p-cysN-f/r, D6-p-cysN-f/r, M4-p-cysN-f/r, and M6-p-cysN-f/r) and restriction enzymes in the *cysN* promoter.

The strains and plasmids used in this study were sourced from the laboratory and are detailed in Table 2. APEC94 was isolated from the intestinal tract of a chicken at a commercial poultry farm. The strains were cultivated in Luria-Bertani (LB) broth (Oxoid, Basingstoke, UK) or on LB solid medium containing 2.0% agar (Oxoid, Basingstoke, UK) at 37°C with aeration. As required, antibiotics, such as ampicillin (100 µg/mL), kanamycin (50 µg/mL), spectinomycin (16 µg/mL), or chloramphenicol (16 µg/mL), were added to the medium. Briefly, the expression and purification of the LsrR protein were determined according to the protocol described by Pillai-Kastoori L. et al. and Liu X. (50, 51). Moreover, the plasmids pcysND4, pcysND6, pcysNM4, and pcysNM9 had mutated promoter regions (four or six nucleotide bases deletion or mutation) of pPcysN (Fig. 12). Briefly, the site mutation was done by following the protocol of the QuickMutation Gene Random Mutation Kit (Beyotime, China), which introduces random mutation into the target gene by error-prone PCR and a thermostable DNA polymerase. The RandomMut DNA polymerase was used for error-prone PCR when the recommended dose of mutation enhancer was added, resulting in mutation rates of 7.8 mutations per kilobase. The AT→GC and GC→AT transition mutations are roughly balanced (AT→GC/GC→AT ratio = 1.06), indicating that the RandomMut DNA polymerase is a completely unbiased error-prone enzyme, and the appropriate number of insertions and deletions can also be introduced.

**TABLE 2:** Strains and plasmids used in this study

Strain or plasmid	Description	Reference or source
Strains		
DH5α competent cells	Clone host strain, *lac*Z∆M15, *rec*A1 mutation, high yield, and quality of DNA due to the *end*A mutation	Thermo Scientific
*E. coli* BHT101	Expression strain, hybrid K12 xB bacterium, recA13 mutation, hsdS20 (rB-MB-) restriction minus genotype, compatible with pUT18C, and pKT25	Laboratory Stock
APEC94	Wild-type isolates from clinical liver samples, avian pathogenic *E. coli* (APEC) wild type	Laboratory Stock
lasmids		
pET-28a	Bacterial expression, promoter *tac,* high (activated with IPTG), transient, Amp^r^, nonviral	Amersham
pUT18C	V*ector* for a bacterial two-hybrid system, T18 fragment (375 bp), lac promoter, Amp^r^	Laboratory
pKT25	V*ector* for a bacterial two-hybrid system, T25 fragment (375 bp), *Cya* gene, lac promoter, kan^r^	Laboratory
pTarget	Mammalian expression, Amp^r^, Spec^r^, transient, nonviral, ORF frame 3, *lac* promoter	Laboratory
pCAS	Constitutive expression of Cas9 and inducible expression of λ red and sgR, Cm^r^ resistance gene, and growth strain DH5α	Laboratory
pSTV28	Bacterial expression, bacterial cloning vector with a p15A origin, *lac* promoter, Cm^r^ resistance gene	Laboratory
pKT25-cysN	V*ector* for a bacterial two-hybrid system, T25 fragment (720 bp), *Cya* gene, lac promoter, kan^r^	This study
pKT25- cysND4	Mutational pKT25-cysN with 4 bp “TCCG” deletion	This study
pKT25- cysND6	Mutational pKT25-cysN with 6 bp “GGAAAA” deletion	This study
pKT25-cysNM4	Mutational pKT25-cysN with 4 bp mutation “TCCG” to “CTTA”	This study
pKT25-cysNM6	Mutational pKT25-cysN with 6 bp mutation “GGAAAA” to “AAGGGG”	This study

### LsrR and *cysN* interaction

Following LsrR expression, the recombinant plasmids pUT18C*-*lsrR and pKT25*-*cysN were constructed using the CRISPR/Cas9 gene-editing technology (52, 53) with some modifications. In brief, pUT18C-lsrR (10 µL) was electrotransformed into the bacterial culture BHT101 (100 µL) using Gene Pulser X-cell modular electroporation systems. After overnight culturing and subsequent centrifugation, reconstitution, and detection by PCR for the appropriate size, the recombinant plasmid pKT25-cysN (10 µL) was electrotransformed into the pUT18C-LsrR-BHT101 (100 µL). Following overnight culturing, centrifugation, reconstitution, and PCR detection to confirm the sequence, X-GAL Luria-Bertani (LB) agar (Oxoid, Basingstoke, UK) plates were prepared by adding X-GAL (20 mg/mL). Then, 300 µL of culture was spread on the X-GAL plates and incubated at 30°C for 24 hours. The *zip* gene was used as a positive control, interacting with protein LsrR, while *ompA* served as a negative control. After incubation, the color of the colonies or growth was observed to indicate LsrR protein interaction with *cysN*.

### Electrophoretic mobility shift assay (EMSA)

EMSA determined the LsrR-*cysN* interaction as previously mentioned (54). In brief, the nucleic acid (155 bp) restriction fragment from the *cysN* promoter-operator region and the protein LsrR were validated, expressed, and purified to a concentration of 1 µg/µL. The concentrations of labeled DNA (0.1 pmol) and untagged DNA (10 pmol) were employed. The Light Shift TMR EMSA Optimization and Control Kit (Thermo Fisher) was used to prepare the EMSA binding reaction following the manufacturer’s procedure. TBE buffer 5× (0.5 mL), ddH_2_o (8.1 mL), 39:1 acrylamide/bisacrylamide 40% wt/vol (1 mL), 80% glycerol (312.5 µL), 10% ammonium persulfate (75 µL), and TIMID (5 µL) were added to a sterilized tube, gently mixed, and incubated at room temperature. An empty EMSA PAGE gel was then run along a 4% native PAGE gel for 20 min. Then, 2 µL of 5× loading buffer and 20 µL of sample were loaded in the well for 90 min at 100 V at 4°C. The lens was focused to 1.5 mm using an Odyssey infrared two-color laser imager to scan the gel on a glass plate. The fluorescein labeling conceals the reaction mixture from light throughout the process.

### DNase I footprinting assay

PCR primer was synthesized and subsequently 5′-labeled with Cy5.5 by the manufacturer (Invitrogen), resulting in the labeled primer p-cysN-f-cy5.5. Using labeled p-cysN-f-cy5.5 and unlabeled p-cysN-r as primers, *E. coli* APEC94 genomic DNA was used as a template to create the labeled DNA fragments by PCR. PAGE was used to purify the identified DNA fragments. DNase I footprinting experiments were conducted using an adapted protocol based on earlier research, employing an Applied Biosystems 3730XL DNA analyzer (55, 56). In the DNA footprinting assay, 50 µL of incubation buffer (50 mM Tris-HCl, pH 7.5, 150 mM NaCl, 3 mM magnesium acetate, 0.1 mM EDTA, and 0.1 mM DTT) was added to the labeled promoter fragments (1 pmol) and incubated for 20 min at room temperature. After that, a final volume of 100 µL of the Ca^2+^/Mg^2+^ solution (50 µL, 5 mM CaCl_2_, and 10 mM MgCl_2_) was added to the mixture and incubated for 5 min at 25°C. The addition of 0.6 U RQ1 RNase-free DNase I (Promega) started the digestion of the DNA. The digest reaction was stopped with the addition of 90 µL of stop solution (200 mM NaCl, 30 mM EDTA, and 1% SDS) after it had been digested for one minute. Following extraction with 200 µL of phenol-chloroform-isoamyl alcohol (25:24:1), the final reaction mixtures were purified using the ethanol precipitation technique. A 3730XL DNA analyzer (Applied Biosystems) was used to load samples for CE and detection. The electropherograms were aligned with GeneMapper 3.5 (Applied Biosystems).

### Growth curve, bacterial motility, and biofilm formation assays

Bacterial growth was measured as described previously (57). Briefly, overnight cultures of APEC94*,* APEC94∆cysN, and cAPEC94∆cysN strains were diluted in fresh LB broth (1:100 [vol/vol]) and incubated at 37°C with rotation at 200 rpm. The OD_600nm_ value of each strain was monitored at 1-hour intervals, and the values were recorded and analyzed to plot each strain’s growth curve. Bacterial motility halos on LB soft agar motility plates (0.5% agar) were assessed following 12 h of incubation at 37°C. Bacterial biofilms were measured in 96-well polyvinyl chloride (PVC) microplates using a crystal violet assay, as described previously (58). Each strain’s overnight culture was diluted 1:100 in Mueller-Hinton Broth (MH Broth) (Thermo Scientific, Oxoid, UK), supplemented with 0.5% glucose (wt/vol) and inoculated into 96-well PVC microplates. Biofilms were allowed to develop at 37°C for 24 h. The cells that adhered to the microtiter wells were stained with crystal violet (0.1% wt/vol), subsequently solubilized with 95% ethanol, and measured at OD_595nm_. Each strain was analyzed in three replicates.

### Bacterial persistence, adhesion, and invasion assays

The assessment of bacterial adhesion and invasion by the APEC94, APEC94∆cysN, and cAPEC94∆cysN strains in RAW 264.7 cells was performed following previously established protocols (59). Briefly, RAW 264.7 cells were cultured in a 24-well plate (Corning Inc., Corning, NY, USA) using DMEM supplemented with 10% FBS until they reached approximately 80% confluence. Each well was then exposed to bacteria at a multiplicity of infection (MOI) of 50. The coculture of cells and bacteria was maintained at 37°C in a 5% CO_2_ atmosphere for 2 h. After this incubation, the medium was discarded, and the wells were washed three times with PBS (pH 7.4). To lyse the RAW 264.7 cells, 0.2% Triton X-100 was used, followed by serial dilution of the lysates and plating on LB agar. Bacterial colonies were counted after 18 h of incubation. APEC-infected RAW 264.7 cells for the invasion assay were treated with DMEM containing gentamicin (100 µg/mL) for 1 h to eliminate extracellular bacteria. Subsequently, the cells were washed and lysed, and the invasive bacteria were quantified by plating on LB agar plates (60, 61). RAW 264.7 cells were infected with APEC strains at an MOI of 50 to evaluate bacterial persistence, and colony-forming units (CFU) were counted at 6, 12, 24, and 48 h post-infection. The experiment was conducted in triplicate, and the mean CFU at each time point was calculated to assess bacterial persistence within the cells (62).

### Serum bactericidal assay

Bacterial resistance to serum bactericidal activity was assessed following previously established protocols, with certain modifications (59). Briefly, bacterial cultures were exposed to different dilutions of SPF chicken serum (12.5%, 25%, 50%, 75%, and 100%) at 37°C for 30 min. Heat-inactivated serum served as a negative control. SPF serum was collected from 6-week-old chickens. The number of surviving bacteria was determined by plating on LB plates.

### Antibiotic susceptibility test (AST) and minimum inhibitory concentration (MIC) assays

Briefly, APEC strains were standardized using the colony suspension method. Each strain’s suspension was matched with 0.5 McFarland standards to give a resultant concentration of 1.5 × 10^6^ CFU/mL. The zone of inhibition (mm) for APEC94 and the mutant strain APEC94∆cysN was determined by using the agar disk diffusion assay according to the modified Kirby-Bauer disk diffusion technique (53, 63), which involved swabbing the Mueller-Hinton agar (MHA) (Oxoids UK) plates with the adjusted overnight culture of each APEC strain. Antibiotic disks were obtained from Oxoid (Basingstoke, UK). Disks containing different antibiotics were aseptically placed on the inoculated agar plates and incubated at 37°C for 24 h. After incubation, the plates were examined for inhibition zones. The diameter of the inhibition zones produced by each antibiotic disk was measured to the nearest millimeter, recorded, and interpreted using the Clinical and Laboratory Standards Institute Zone Diameter Interpretative Standards (63). Similarly, the MIC was performed on strains according to EUCAST recommendations (64). In brief, APEC94, APEC94∆cysN, and cAPEC94∆cysN strains were subcultured on MH agar (Oxoids UK) plates and incubated overnight at 37°C. The strains were added to PBS (2 mL) until a 0.5 (0.4–0.6) McFarland turbidity standard was achieved, equivalent to 1.5 × 10^8^  CFU/mL, as determined using a densitometer (DensiCHECK, Biomerieux). Then, 50  µL was added to 11.5  mL of MH Broth (Sigma Aldrich, Missouri, USA). A volume of 100  µL was added to each well of the 96-well microtiter plate, achieving a final inoculum concentration of 5 × 10^5^  CFU/mL in the well. The plate was incubated at 37°C for 20 h and then observed by ocular inspection, comparing the results with the established breakpoints for *E. coli* by EUCAST (64). The MIC was determined as the lowest solution concentration capable of visually inhibiting 100% of the APEC94 strain growth. The zone of inhibition (mm) was classified as susceptible (S), intermediate (I) (susceptible to increased exposure), and resistant (R) to antibiotics, according to the zone diameter interpretation standard recommended by the Clinical Laboratory Standards Institute (53).

### Molecular characterization of efflux pump gene expression (qRT-PCR)

The APEC94, APEC94ΔcysN, and cAPEC94ΔcysN strains were cultured in Luria-Bertani (LB) broth at 37°C with various antibiotics (Table 3). Bacterial cultures were grown to an optical density of 0.6 at OD_600nm_ before RNA extraction. Total RNA was extracted using TRIzol reagent (Invitrogen, USA), according to the manufacturer’s protocol. Residual genomic DNA was removed by DNase I treatment (Thermo Fisher Scientific), and RNA integrity was confirmed using 1% agarose gel electrophoresis. Complementary DNA (cDNA) was synthesized from 1 µg of purified RNA with the PrimeScript RT Reagent Kit (Takara, Japan). qRT-PCR was conducted using the SYBR Green Master Mix (Takara, Japan) on a CFX96 Real-Time PCR Detection System (Bio-Rad, USA). The primers targeting the efflux pump genes (*acrA* and *tolC*) and the housekeeping gene (16S rRNA) were designed using Primer3 software. Each qRT-PCR reaction was conducted in a final volume of 20 µL, comprising 10 µL SYBR Green Master Mix, 0.4 µL of each primer (10 µM), 2 µL of cDNA, and 7.2 µL of nuclease-free water. The cycling conditions were as follows: initial denaturation at 95°C for 3 min, followed by 40 cycles of 95°C for 10 s, 60°C for 30 s, and 72°C for 30 s. Relative gene expression was calculated using the 2^(-ΔΔCt) method, with 16S rRNA as the internal control. Each experiment was performed in triplicate, and the mean values were reported with standard deviations.

**TABLE 3:** The susceptibility of different antibiotics to APEC94, mutant, and complementary strain

Class of antibiotics	Drug (μg)	Interpretive criteria (mm)	APEC94	APEC94∆cysN	cAPEC94∆cysN
Lincosamides	Clindamycin (CLI-2)	(R ≤ 14, I 15–20, S ≥ 21)	R	S	S
Carbapenems	Meropenem (MEM-10)	(R ≤ 13, I 14–15, S ≥ 16)	S	S	S
Cephalosporins	Ceftazidime (CAZ-30)	(R ≤ 17, I 18–20, S ≥ 21)	S	S	S
	Cefepime (FEP-30)	(R ≤ 14, I 15–17, S ≥ 18)	S	S	S
Amphiphenicols	Florfenicol (FFC-30)	(R ≤ 14, I 15–19, S ≥ 20)	R	S	S
Penicillin	Ampicillin (AMP-10)	(R ≤ 13, I 14–16, S ≥ 17)	R	R	S
	Amoxicillin (AMC-10)	(R ≤ 13, I 14–16, S ≥ 17)	S	S	S
Aminoglycosides	Kanamycin (KAN-15)	(R ≤ 13, I 14–17, S ≥ 18)	R	S	S
	Streptomycin (STR-10)	(R ≤ 11, I 12–14, S ≥ 15)	R	R	S
Tetracycline	Tetracycline (TCY-30)	(R ≤ 14, I 15–18, S ≥ 19)	S	I	S
Polypeptide	Polymyxin B (PB-30)	(R ≤ 8, I 9–11, S ≥ 12)	S	S	S
Co-trimoxazole	Sulfamethoxazole/trimethoprim (SXT-25)	(R ≤ 10, I 11–15, S ≥ 16)	S	R	S
Fluoroquinolone	Enrofloxacin (ENR 5)	(R ≤ 13, I 14–16, S ≥ 17)	S	S	S

### RNA isolation and quantitative real-time PCR (qRT-PCR)

The expression of virulence genes in the APEC94 strains and the mRNA levels of inflammatory cytokines in infected mouse tissues (heart, lung, liver, spleen, and kidney) were quantified by qRT-PCR, following established protocols (65). For bacterial gene expression, APEC94, APEC94∆cysN, and cAPEC94∆cysN strains were harvested during the logarithmic growth phase (59). For all samples, total RNA was extracted using TRIzol reagent (Invitrogen). Contaminating DNA was removed using a Turbo DNA-free Kit (Thermo Fisher), and cDNA was synthesized from 1 µg of RNA using the PrimeScript RT Reagent Kit (TaKaRa). qRT-PCR was performed using SYBR Premix Ex Taq (TaKaRa) on a CFX96 Real-Time PCR Detection System (Bio-Rad). The 20 µL reaction mixture consisted of 10 µL of SYBR Green Master Mix, 0.4 µL of each gene-specific primer (10 µM, as listed in Table 1), 2 µL of cDNA, and 7.2 µL of nuclease-free water. The thermal cycling protocol consisted of the following steps: 95°C for 3 min, followed by 40 cycles of 95°C for 10 s, 60°C for 30 s, and 72°C for 30 s. A melt curve analysis was performed to confirm amplification specificity. Relative gene expression was calculated using the 2–ΔΔCt method (66), normalized to the *dnaE* gene for bacterial transcripts or the *β-actin* gene for murine cytokines. PCR efficiency for each gene was confirmed to be >90% via standard curves. All assays were run in duplicate with three independent biological replicates. The efficiency of PCR for each gene was confirmed through standard dilution curves, achieving efficiencies greater than 90%. Each assay was conducted in duplicate and repeated three times.

To assess the transcriptional regulation of *cysN* by LsrR, the APEC94 and the APEC94ΔlsrR mutant strains were grown in LB broth at 37°C to mid-logarithmic phase (OD_600nm_ = 0.6). RNA extraction, cDNA synthesis, and qRT-PCR were performed as described above. The relative expression of *cysN* was determined using the 2–ΔΔCt method with *dnaE* as the internal control and the APEC94 strain as the calibrator.

### Animal infection experiments

All animal experiments were conducted following the Guidelines on the Humane Treatment of Laboratory Animals by the Ministry of Science and Technology of the People’s Republic of China and were approved by the Institutional Animal Care and Use Committee at the Shanghai Veterinary Research Institute (Ethics Approval Number: SV-20231229-Go5). The mice were used to assess the pathogenicity of the APEC94, APEC94∆cysN, and cAPEC94∆cysN strains. APEC strains were harvested, washed twice in PBS, and dose-corrected during the exponential phase. A total of 32 mice were divided into four groups, each containing eight male C57/black 6-week-old mice purchased from Jiangsu Huachuang Sino Pharma Tech Co., Ltd., Taizhou City, Jiangsu Province, China, and given either 10^6^ colony-forming units (CFUs) of bacteria intraperitoneally or PBS as a negative control. Up to seven days after the infection, survival and fatality rates were monitored daily. Similarly, a mouse model was used for the systemic infection experiment to investigate the impact of *cysN* on APEC94 colonization *in vivo* (19, 67). In brief, a group of eight male C57/black mice, 6 weeks old, was infected intraperitoneally with a bacterial suspension containing 10^8^ colony-forming units (CFUs) and was humanely euthanized 24 h post-infection. The detection limit was 100 CFU/tissue. The blood, heart, lung, liver, spleen, and kidney were collected, weighed, and homogenized. Serial dilutions of the homogenates were plated onto LB plates to count the bacterial numbers. The colonization and proliferative capacity of the APEC strain in the blood, heart, lung, liver, spleen, and kidney were compared (68).

### Cytokine quantification by ELISA

Cytokine levels (IL-2, IL-4, IL-6, IL-10, TNF-α, and IFN-γ) in serum and tissue homogenates (lung, heart, liver, spleen, and kidney) were quantified using the MILLIPLEX Mouse Cytokine/Chemokine Magnetic Bead Panel—Premixed 32 Plex—Immunology Multiplex Assay (Millipore Sigma, Burlington, MA, USA). This assay utilizes the Luminex xMAP technology, enabling simultaneous detection of multiple analytes. Tissue samples (lung, heart, liver, spleen, and kidney) were harvested, rinsed with phosphate-buffered saline (PBS), and homogenized in ice-cold lysis buffer containing protease inhibitors. The homogenates were centrifuged at 12,000 × *g* for 15 min at 4°C, and the supernatants were collected for cytokine analysis. A volume of 25 µL of each sample was incubated with magnetic beads pre-coated with specific capture antibodies in a 96-well plate. Following incubation, the plate was washed, and detection antibodies were added, followed by streptavidin-phycoerythrin. Plates were analyzed using the Luminex MAGPIX system, and cytokine concentrations were calculated from standard curves generated using known cytokine concentrations. All procedures were performed following the manufacturer’s protocol, ensuring reproducibility and accuracy.

### Statistical analysis

The results were analyzed using GraphPad Prism 5.0 software (GraphPad Software Inc., La Jolla, CA, USA). All experiments were performed in biological triplicates (*n* = 3), and differences were analyzed using one-way ANOVA with Tukey’s post hoc test, two-way ANOVA with Bonferroni correction, or Mann–Whitney U test for *in vivo* data, with significance considered at a *P* value of < 0.05. Data are expressed as means and standard errors of the means (SEM) from three independent experiments.

## Data Availability

The data sets generated for this study are available at Doi: (10.17632/tgjh6mwhwz.1), (10.6084/m9.figshare.28409531).

## SUPPLEMENTAL MATERIAL

Supplemental material iai.00421-25-s0001.docx Fig. S1 to S5.

ASM does not own the copyrights to Supplemental Material that may be linked to, or accessed through, an article. The authors have granted ASM a non-exclusive, world-wide license to publish the Supplemental Material files. Please contact the corresponding author directly for reuse.
