# Supplementary material for: Quorum-sensing regulator LsrR modulates avian pathogenic Escherichia coli pathogenicity through direct regulation of cysN
Source: Infect Immun. 2025 Nov 28;94(1):e00421-25. doi: 10.1128/iai.00421-25 (PMC12798060; doi:10.1128/iai.00421-25)
Supplement: Supplemental material — Fig. S1 to S5. [file iai.00421-25-s0001.docx]

Clindamycin (CLI-2 μg), Meropenem (MEM-10 μg), Ceftazidime (CAZ-30 μg), Cefepime (FEP-30 μg), Florfenicol (FFC-30 μg), Ampicillin (AMP-10 μg), Amoxicillin/Clavulanate (AMC 20 μg/10 μg), Kanamycin (KAN-15 μg), Streptomycin (STR-10 μg), Tetracycline (TCY-30 μg), Penicillin B (PB-30 μg), Sulfamethoxazole/ Trimethoprim (SXT-25 μg), and Enrofloxacin (ENR 5 μg).

**Supplementary Fig. 1: (a)** ZOI after the MH agar plate incubation for mutant APEC94∆cysN strain. (b) ZOI of different antibiotics after incubation of the MH agar plate for APEC94. (c) ZOI after the MH agar plate incubation for the mutant cAPEC94∆cysN strain. (d) ZOI of different antibiotics after incubation of the MH agar plate for ATCC25592. The marked antibiotics showed alteration in different bacterial strains.

**Supplementary Fig. 2:** qRT-PCR Analysis of efflux pump Gene expression (*acrA* and *tolC*). Relative mRNA expression of efflux pump genes (*acrA* and *tolC*) in APEC94, APEC94ΔcysN, and cAPEC94ΔcysN under different antibiotic treatments. Gene expression was normalized to 16S rRNA, and the fold change was calculated using the 2^(-ΔΔCt) method. Data are presented as mean ± standard deviation (SD) of three independent experiments (n=3). Statistical significance is indicated (**p* < 0.05, ***p* < 0.01, ****p* < 0.001). (a) Streptomycin (STR-10), (b) Clindamycin (CLI-2), (c) Meropenem (MEM-10), (d) Ceftazidime (CAZ-30), (e) Cefepime (FEP-30), (f) Florfenicol (FFC-30), (g) Ampicillin (AMP-10), (h) Amoxicillin (AMC-10), (i) Sulfamethoxazole/Trimethoprim (SXT-25).

**Supplementary Fig. 3:** Transcription levels of IL-2, IL-4, and IL-10 in tissues from mice infected with APEC strains. Tissues were collected and analyzed as in Fig. 11. The transcription levels of (a) IL-2, (b) IL-4, and (c) IL-10 are shown. Statistical significance was assessed by two-way ANOVA (****p* < 0.001, ***p* < 0.01, **p* < 0.05, ^ns^*p* > 0.05).

**Supplementary Fig. 4:** (a) The plasmids with cysN promoter regions, including 4- or 6-bp nucleotide base deletions or mutations, were derived from the promoter plasmid pPcysN. The spaces represent deletions, while the brown squares represent random GC replacements. (b) EMSA of the site deleted the cy5.5-labeled *cysN* probe from the LsrR protein. (c) EMSA of site-mutated cy5.5-labeled *cysN* probe to LsrR protein.

**Supplementary Fig. 5:** LsrR specifically binds to the *cysN* promoter region. EMSA demonstrating the specific binding of purified LsrR protein to a fluorescently labeled *cysN* promoter fragment. Lane 1: 50 fmol Cy5.5-labeled *cysN* promoter probe (p-cysN), 6 µM LsrR, and 200 fmol unlabeled specific competitor (unlabeled p-cysN). Lane 2: 50 fmol Cy5.5-labeled non-specific competitor probe (p-ompA) and 6 µM LsrR. The presence of a shifted band (B) in Lane 1 indicates the formation of a stable protein-DNA complex between LsrR and the p-*cysN* probe. The absence of a shifted band in Lane 2 confirms that the observed binding is specific to the *cysN* promoter sequence and not a non-specific interaction with DNA. The unbound free DNA is indicated by (F). Lane: 3 free probes. Lane 4-5: 200 fmol of unlabeled probes were incubated with the LsrR protein beside the labeled probes. In lane 5: protein FabH was run as a negative control and showed no effect on the binding. Cy5.5-p-ompA was run as a control for DNA to ensure Specific binding.
